# *ARID1A* deficiency weakens BRG1-RAD21 interaction that jeopardizes chromatin compactness and drives liver cancer cell metastasis

**DOI:** 10.1038/s41419-021-04291-6

**Published:** 2021-10-23

**Authors:** Xue-Ying Shang, Yi Shi, Dan-Dan He, Lan Wang, Qing Luo, Chuan-Huai Deng, Yu-Lan Qu, Na Wang, Ze-Guang Han

**Affiliations:** Key Laboratory of Systems Biomedicine (Ministry of Education), Shanghai Center for Systems Biomedicine, Shanghai Jiao Tong University, Shanghai, 200240 China

**Keywords:** Metastasis, Chromatin remodelling

## Abstract

*ARID1A*, encoding a subunit of SWI/SNF chromatin remodeling complex, is widely recognized as a tumor suppressor gene in multiple tumor types including liver cancer. Previous studies have demonstrated that *ARID1A* deficiency can cause liver cancer metastasis, possibly due to the altered chromatin organization, however the underlying mechanisms remain poorly understood. To address the effect of *Arid1a* deficiency on chromatin organization, we generated chromatin interaction matrices, and exploited the conformation changes upon *Arid1a* depletion in hepatocytes. Our results demonstrated that *Arid1a* deficiency induced A/B compartment switching, topologically associated domain (TAD) remodeling, and decrease of chromatin loops. Further mechanism studies revealed that ATPase BRG1 of SWI/SNF complex could physically interact with RAD21, a structural subunit of chromatin architectural element cohesin; whereas *ARID1A* deficiency significantly diminished the coupled BRG1-RAD21. Interestingly, the tumor-associated genes within the switched compartments were differentially expressed depending upon *Arid1a* depletion or not. As a consequence of *ARID1A* deficiency-induced conformational alteration, the dysregulation of some genes such as *PMP22* and *GSC*, promoted the invasion capacity of liver cancer cells. This study provides an insight into liver cancer tumorigenesis and progression related to *ARID1A* mutations.

## Introduction

SWI/SNF complex is an evolutionarily conserved multi-subunit machine that remodels nucleosome structure to modulate transcription by sliding and catalyzing the inclusion and ejection of histone octamers in an ATP-dependent manner [1]. Some genes encoding the subunits of the SWI/SNF complex were found to be frequently mutated in approximately 20% of human cancers, indicating that the abnormal complex plays a significant role in cancer initiation and progression [2]. Among these genes, *ARID1A* has the highest mutation frequency in many tumors, and is widely recognized as a tumor suppressor owing to the extensive loss-of-function mutations [3, 4]. Our previous work demonstrated that *ARID1A* was mutated in 13% of hepatitis B virus (HBV) infection-associated hepatocellular carcinoma (HCC) specimens, and *ARID1A* mutations were considered as a crucial event in liver cancer metastasis [5]. Nevertheless, the underlying mechanisms that *ARID1A* deficiency-mediated metastasis remain poorly understood.

The eukaryotic chromatin is orderly involved in DNA replication and transcription during development, which determines diverse cell-type differentiation owing to the hierarchically efficient chromatin organization within three-dimensional (3D) nuclear space [6]. Chromatins occupy a discrete territory in the nucleus, and have been revealed to preserve three folding paradigms: compartments, topologically associated domains (TADs) and chromatin loops [7]. Genomic regions are spatially segregated into active and repressive chromatin domains termed compartments A and B, respectively [8]. Compartmentalization is a dynamic process switching in differentiation of human embryonic stem cells [9] and tumorigenesis [10]. At the sub-megabase scale, TADs are aggregated by higher density interactions, and insulated from neighboring regions by boundaries that are enriched with cohesin complex and CCCTC-binding factor (CTCF) in a convergent manner [11, 12]. Chromatin loops, the contact pairs with closer spatial proximity, are the basic structural and functional units in higher-order genome organization, which are formed by CTCF anchoring and cohesin-driven extrusion [13]. The cohesin complex comprises four conserved subunits, of which the highly extended protein α-kleisin RAD21 tethers V-shaped SMC1/3 heterodimer and recruits STAG to the complex [14]. Whether and how SWI/SNF complex could function as an architectural cooperator to synergistically stabilize chromatin organization with CTCF or cohesin complex should be explored, especially in tumorigenesis.

Accumulating evidences have illustrated that higher-order genome disorganizations are associated with the process of normal cells progressively evolving to a neoplastic state; examples can be found in prostate cancer [15], gastrointestinal stromal tumors (GISTs) [16], gliomas [17], and hematologic malignancies such as T cell acute lymphoblastic leukemia (T-ALL) [18]. Thus, an in-depth investigation on chromatin organization is vitally important for disclosing the underlying mechanism of tumorigenesis and progression in *ARID1A*-mutated liver cancer.

Intriguingly, the distinct roles of the core subunit ATPase BRG1 and ARID1A in genome organization have been reported in two types of cancer cells. BRG1 is crucial for telomere organization and TAD boundary maintaining in proliferating mammary epithelial cells [19], while ARID1A contributes to B-compartment formation and weakening of TAD border strength in ovarian clear cell carcinoma (OCCC) cells [20]. It is doubtless that SWI/SNF complex is involved in the organization of chromatin conformation, however, the functions of different subunits of the complex could be completely distinct because of different cell types. To explore the precise role of *ARID1A* mutations in higher-order genome reorganization of hepatocytes, in this work, we attempt to reveal the conformational changes induced by *ARID1A*-deficiency, and then define the liver cancer metastasis-related genes that arose from the conformational remodeling. This study would provide a novel insight to the *ARID1A* deficiency-driven liver cancer metastasis due to the chromatin organization change.

## Materials and methods

### Cell culture

AB17 cells were immortalized primary hepatocytes isolated from *Arid1a*^*fl/fl*^ mouse liver, successively infected by the simian virus 40 large T antigen (SV40LT) and pLPC-H-Ras V12D viruses and screened by puromycin. Q506 cells were also immortalized *Arid1a*^*fl/fl*^ primary hepatocytes while only infected by SV40LT virus for eliminating the experimental impacts pLPC-H-Ras V12D virus brought. Mouse embryo fibroblast (MEF) cells were isolated from E13.5 mouse embryos with *Arid1a*^*fl/fl*^ genotype. To obtain the *Arid1a* wildtype (WT) and knockout (KO) cells, AB17, Q506, and MEFs were infected by Ad-GFP and Ad-Cre virus respectively. Mouse cells and human cell lines HEK293T, MHCC-97H, SK-HEP-1, and HepG2 were cultured in Dulbecco’s Modified Eagle Medium (DMEM), and human endometrial cancer cell lines Ishikawa (ISK) and HEC-1-A were maintained in DMEM/F12, all were supplemented with 10% fetal bovine serum (FBS) and 1% penicillin/streptomycin at 37 °C and 5% CO_2_. All cell lines have been tested with no mycoplasma contamination.

### Plasmids and transfection

The plasmids encoding T7-ARID1A (CMV-T7-hOsa1, #17986) and BRG1-FLAG (pCMV5 BRGI-Flag, #19143) were purchased from Addgene, of which CMV-T7-hOsa1 was reconstructed on account of 5’ end missing. The full length of *CTCF* and *RAD21* coding sequences were cloned into eukaryotic expression vector pcDNA3.1(+)-HA. For RNA interference experiments, shRNAs were inserted to the pLKO.1-puro plasmid. *ARID1A*-null HCC cell line was established using CRISPR/Cas9 that sgRNA was inserted to lentiCRISPRv2 plasmid and co-transfected HEK293T cells with the packaging vectors pVSVg and psPAX2. Transfection assays were performed using Lipofectamine 2000 (Invitrogen) according to the manufacturer’s protocol. Viral supernatants were collected 48 hr post-transfection and filtered through a 0.45 μm filter. The information of shRNA and sgRNA sequences were listed in Table S1.1.

### Mouse experiments

Hepatocyte-specific *Arid1a* knockout (*Arid1a*^*LKO*^) C57BL/6 mice were generated by mating *Arid1a*^*fl/fl*^ mice (kindly provided by Zhong Wang at the Cardiovascular Research Center, Massachusetts General Hospital, Harvard Medical School) with Albumin-Cre (*Alb-Cre*) mice (from the Jackson Laboratory). The littermate *Arid1a*^*fl/fl*^ mice were served as controls. The *Arid1a*^*fl*^ allele enables to delete the eighth exon of *Arid1a* in the presence of Cre recombinase, leading to a frameshift mutation and nonsense-mediated mRNA decay [21]. Five- to 7-week-old *Ubc-CreER*^*T2*^; *Arid1a*^*fl/fl*^ C57BL/6 mice and littermate control *Arid1a*^*fl/fl*^ mice were administered 1 mg of tamoxifen (T5648, Sigma) dissolved in sunflower oil via intraperitoneal injection for five consecutive days. All animals were randomly grouped. Mice genotype identification was as previously described [21].

### Western blotting and co-immunoprecipitation assay

Cells and mice tissues were lysed in ice-cold protein extraction buffer [50 mM tris (pH 7.4), 150 mM NaCl, 1 mM EDTA, 1% Triton X-100, 50 mM NaF, 10 mM sodium pyrophosphate, 10 mM sodium β-glycerophosphate] with protease inhibitor cocktails (Roche) and quantified using the BCA Protein Assay Kit (ThermoFisher). The lysates were immunoblotted against primary antibodies as follows: ARID1A (Cell Signaling Technology, 12354S), BRG1 (Santa Cruz Biotechnology, sc-17796), BAF170 (Bethyl Laboratories, A301-039A), BAF53A (Bethyl Laboratories, A301-391A), SNF5 (Santa Cruz Biotechnology, sc-13055), CTCF (Cell Signaling Technology, 3418S), RAD21 (Abcam, ab992), SMC3 (Abcam, ab9263), HA tag (Abcam, ab18181), Flag tag (Abcam, ab49763), T7 tag (Abcam, ab9138), PMP22 (Santa Cruz Biotechnology, sc-515199), GSC (R&D Systems, AF4086) and β-actin (Sigma, A5441). The blots were visualized with peroxidase-coupled secondary antibodies. Co-immunoprecipitation (Co-IP) assay was conducted with the same antibodies. Briefly, nuclear extracts were diluted with lysis buffer to a final concentration of 1 mg/ml and incubated with 2 µg of antibody on a rotator overnight. Protein G beads (Millipore) were added and incubated for 3 h. Then the beads were washed with 1 ml hypersaline lysis buffer (300 mM NaCl) for 5 times. Finally, beads were resuspended in SDS loading buffer and analyzed by immunoblotting.

### In vitro binding assays

BRG1 was synthesized in vitro using T_N_T Quick Coupled Transcription/Translation Systems (Promega) as specified by the manufacturer. Briefly, 1 μg of BRG1-Flag expressing vector with T7 promoter was incubated with 40 μl of T_N_T master mix and 1 μl of 1 mM methionine at 30 °C for 90 min. The Glutathione S-transferase (GST) and GST-RAD21 was expressed in Transetta (DE3) Chemically Competent Cells induced overnight at 20°C. For in vitro interaction studies, the purified GST and GST-RAD21 proteins were incubated with BRG1-Flag in binding buffer at 4 °C for 2 h with protease inhibitor cocktails (Roche). Then the equilibrated glutathione-Sepharose beads were added to the mixture for 2 h. The beads were washed five times with binding buffer, resuspended in 50 μl of SDS-PAGE loading buffer, and detected by immunoblotting.

### Immunofluorescence assay

Cells were fixed with 4% paraformaldehyde and permeabilized with 0.2% Triton X-100 in PBS. Primary antibodies were applied at the following dilutions: BRG1 (Santa Cruz Biotechnology, sc-17796) and RAD21 (Abcam, ab992). Images were acquired with a Nikon A1Si confocal microscope.

### Glycerol and sucrose gradient sedimentation assay

Nuclear fractions for sedimentation assays were lysed and homogenized in lysis buffer [10 mM HEPES, 2 mM MgCl2, 10 mM KCl, 0.5% NP40, 0.5 mM EDTA, 150 mM NaCl, 1 mM DTT, 1 mM PMSF and protease inhibitor (Roche)] for 10 min on ice with intermittent vortexing. The supernatants were slowly and evenly overlaid onto a 5.5 ml 5 to 35% glycerol gradient or 5 to 50% sucrose gradient prepared in a 5.9 ml quick-seal polyallomer centrifuge tube (Beckman Coulter, 355537) followed by centrifugation in an SW-41Ti swing-bucket rotor for 16 h at 35,000 rpm. Fractions were collected for immunoblotting analysis.

### Immunohistochemistry (IHC)

The liver tissues were deparaffinized, rehydrated, and incubated with 4.5% H_2_O_2_ to block endogenous peroxidase activity, successively. Antigen retrieval was performed with 10 mM sodium citrate buffer (pH 6.0). For GSC staining, the samples were blocked with 5% horse serum at room temperature for 1 h, and incubated with GSC (Sangon Biotech, D264135) antibody at 4 °C overnight and then incubated with the secondary antibody. PMP22 (Santa Cruz Biotechnology, sc-515199) staining was performed using the M.O.M Immunodetection Kit (Vector Laboratories) according to the instruction of the manufacturer. IHC staining results were scored by assessment of both the intensity of staining (negative score = 0, weak score = 1, moderate score = 2, strong score = 3) and the percentage of target positive cells. The IHC score was calculated by multiplying the percentage of target positive cells by the intensity score (*n* = 5 per group). IHC analyses were performed by two independent observers who were blinded to the clinical outcome.

### In vitro invasion assay

Invasion assays were performed using transwell filter chambers (8 μm pores, Corning Life Sciences) according to the manufacturer’s instructions. 2 × 10^4^ cells were suspended in 100 μl serum-free medium and added on the upper chamber coated with matrigel. The 600 μl medium supplemented with 10% FBS was added to the lower chamber as a chemoattractant. The invasive cells were fixed in 0.4% PFA post 24 h, stained with 0.1% crystal violet, and counted by an inverted microscope. The mean values of triplicate independent assays were applied.

### In situ DNase Hi-C

Hi-C libraries of AB17 cells infected by Ad-GFP and Ad-Cre were constructed with two biological replicates by in situ DNase Hi-C employed as the published protocol [22]. Ten million cells were fixed in 1% formaldehyde to reversibly cross-link protein-DNA interactions. Then the fixed cells were lysed in a relatively mild condition with 0.3–0.5% SDS to liberate nuclei and digested by DNase I in the presence of divalent manganese. The digested chromatin was end-repaired and dATP-tailed, facilitating the ligation of ‘bridge’ adaptor containing a single biotinylated thymidine, a half BamHI restriction site, and a four-base overhang. After clearing out excess adaptors, the free ends of the chromatin were phosphorylated by T4 PNK and proximity-ligated in situ with T4 DNA ligase. During all of these steps, nuclei are immobilized against carboxylated paramagnetic beads. Then nuclei proceeded cross-linking reversal, isopropanol precipitation, and DNA purification. The DNA fragments were optionally sheared to a size of 100–500 bp by Covaris sonicator and purified by streptavidin beads, following end-repaired, dA-tailed, and ligated to standard Illumina sequencing adaptors. Finally, ligation products were PCR amplified to generate libraries and digested by BamHI to assess the efficiency of proximity ligation. The libraries were sequenced via the Illumina HiSeq X Ten system. Oligonucleotides for the Hi-C library were listed in Table S1.2.

### RNA-seq and qRT-PCR

RNAs were isolated with TRIzol reagent (Life Technologies) and performed on three independent biological replicates. Extracted RNAs were digested with DNase I and purified using the RNeasy Mini Elute Cleanup Kit (Qiagen). The mRNAs were separated by the NEBNext Poly(A) mRNA Magnetic Isolation Module based on the coupling of Oligo d(T)_25_ to paramagnetic beads which were then served as the solid support for the direct binding of poly(A) + RNA. Sequencing libraries were generated using NEBNext^®^ Ultra™ Directional RNA Library Prep Kit^®^ for Illumina according to the manufacturer’s instructions, and paired-end sequenced by Illumina HiSeq-2500.

For quantitative reverse transcription-polymerase chain reaction (qRT-PCR), cDNA was synthesized with 1 mg of total RNA using PrimeScript^®^ RT reagent Kit with gDNA Eraser (TaKaRa). Gene expression levels were detected with TB Green^®^ Fast qPCR Mix (TaKaRa) and calculated by 2^−ΔΔCt^ method. All genes expression were normalized to GAPDH mRNA level as the internal control. Primer sequences for qRT-PCR were listed in Table S1.3.

### Chromatin immunoprecipitation (ChIP) assay

ChIP assay was performed according to the protocol developed by Upstate Biotechnology. Protein G beads were pre-blocked and conjugated with 2 μg antibodies at 4 °C overnight. The next day, the cells were cross-linked by 1% formaldehyde, lysed, and sonicated with Bioruptor^TM^ UCD-200 in sequence, of which 1.25% cell lysate from each sample were saved as WCE DNA and stored at −20 °C. The lysate was incubated with the antibody-bound beads on the rotator at 4 °C overnight. The beads next were adequately washed for seven times with RIPA buffer avoiding the false-positive fragments residual. Finally, both beads and frozen WCE input DNA were reversely cross-linked and purified using PCR Purification Kit (Qiagen) after RNase A and Proteinase K treatment. The antibodies for ChIP assays were ARID1A (Cell Signaling Technology, 12354S), BRG1 (Santa Cruz Biotechnology, sc-17796), CTCF (Cell Signaling Technology, 3418S), RAD21 (Abcam, ab992), H3K4me1 (Abcam, ab8895), H3K27ac (Abcam, ab4729), H3K9me3 (Abcam, ab8898) and H3K27me3 (Abcam, ab108245). The ChIP libraries for Illumina sequencing were constructed using NEBNext ChIP-Seq Library Prep Master Mix and sequenced on Illumina HiSeq-2500 instrument. The primers information for ChIP-QPCR assays were listed in Table S1.4.

### Chromosome conformation capture

Chromosome conformation capture (3C) was performed as in situ Hi-C protocol with a few modifications [23]. Briefly, cells were lysed in an ice-cold lysis buffer for 15 min. The intact nuclei were resuspended and permeabilized in restriction enzyme buffer containing 0.3% SDS. Then the chromatin was digested by 100U of EcoRI at 37 °C with rotation overnight. The free ends of chromatin were proximity-ligated with T4 DNA ligase at room temperature for 4 h. Following the pellets were resuspended and reverse cross-linked at 65 °C overnight. After RNase A and proteinase K treatment, ligation fragments were purified by phenol/chloroform. Primers for 3C PCR were 30–32 bp in length and positioned within 100 bp of restriction enzyme sites (Table S1.4). 3C analyses were performed in two independent 3C libraries from each cell line. GAPDH promoter was used as a negative control.

### Bioinformatics analysis

#### Hi-C contact matrices generation

We adopted the process introduced in situ DNase Hi-C protocol [22] and our own work [24] to compute Hi-C contact matrices based on paired sequencing reads, in which the paired reads were first mapped to *Mus musculus* reference genome (mm10) reference genome individually using BWA mem option. Then we join the reads which both have mapping on the reference genome. After removing PCR redundancy and self-ligations, the primary matrices were generated. The KRNorm algorithm was further applied to obtain normalized matrices. The Juicebox [25] and the 3D Genome Browser [26] were used for visualizing 3D genome organization.

#### A/B compartment determination

To determine the compartment type of each chromosome loci, by borrowing a similar protocol we developed before [24], we used individual chromosome Hi-C contact maps. We first diagonal normalized each contact map by dividing the contact frequencies by their corresponding off-diagonal mean. Then we computed the correlation coefficient (Pearson) matrices for each chromosome, and the compartment type was jointly determined by the sign of the eigenvector corresponding to the first eigenvalue of the principal component analysis (PCA) and the signal of the epigenetic histone markers H3K4me1 and H3K27ac for active, and H3K9me3 and H3K27me3 for inactive.

#### TAD Identification

We adopted our previously developed method TopDom to identify TADs for each chromosome [24]. In detail, a sliding window with default parameter were employed to calculate binSignal for each bin, the local minima were identified as TAD boundaries after an extra smoothing process for the binSignal.

#### Chromatin loop identification

We used HiCCUPS from Juicer to identify chromatin loops [25]. The default parameters were chosen and the algorithm was performed on Hi-C matrices at 25-kb resolution.

#### Chromatin 3D simulation

We employed molecular dynamics (MD) and developed a 3D conformation modeling approach based on Hi-C data as constraints. The chromatin bins were coarse-grained as beads and the intact genome was represented by bead-on-the-string structures consisting of 21 polymer chains. The beads’ spatial positioning is affected by both chromatin connectivity that constrains linearly neighboring beads in close 3D proximity and chromatin activity that ensures active regions tend to be located closer to the nucleus center. The chromatin activity was determined according to compartment degree that can be directly calculated from Hi-C matrices as described above [27]. Based on the compartment degree index, beads were assigned distance values with respect to the nuclear center; the conformation of chromatin was then optimized from random structures with a molecular dynamics approach by applying bias potential to satisfy these distance constraints. For each cell linage, feasible conformation structures were further optimized from random ones to reduce possible variation for further analysis.

#### RNA-seq data analysis

Raw sequencing reads were trimmed by Trimmomatic (v0.36) to remove adapters and low-quality sequences [28]. The filtered reads were mapped to the mm10 genome by TopHat2, following assembled, counted, and normalized with the Cufflinks [29]. Differentially expressed genes (DEGs) were analyzed by using Cuffdiff in the Cufflinks suite using *p-value* <0.05 and fold change >2 as cutoff. Enrichment analysis on DEGs were conducted via Metascape [30] and Reactome [31].

#### ChIP-seq data analysis

Reads were aligned to the mm10 reference genome using bowtie2 [32]. Raw data were filtered as following: (1) low-quality reads removed by SAMtools [33]; (2) PCR duplicates removed using Picard tool (http://broadinstitute.github.io/picard). The MACS2 with default parameters was deployed for peak calling, with corresponding inputs as background separately [34]. The chromatin immunoprecipitation combined with high-throughput sequencing (ChIP-seq) datasets acquired from the ENCODE database have been listed in Table S2 [35].

### Statistical analysis

The univariate Kaplan-Meier method was used to estimate the cumulative probability of relapse-free survival and overall survival of HCC patients [36]. Unless stated otherwise, data were presented as mean ± SEM and were tested for normality using SPSS Statistics v23. A two-tailed Student’s *t*-test was applied to evaluate statistical variance between groups. *p* value <0.05 was considered statistically significant.

## Results

### *Arid1a* deficiency alters chromatin conformation

**Fig. 1:**
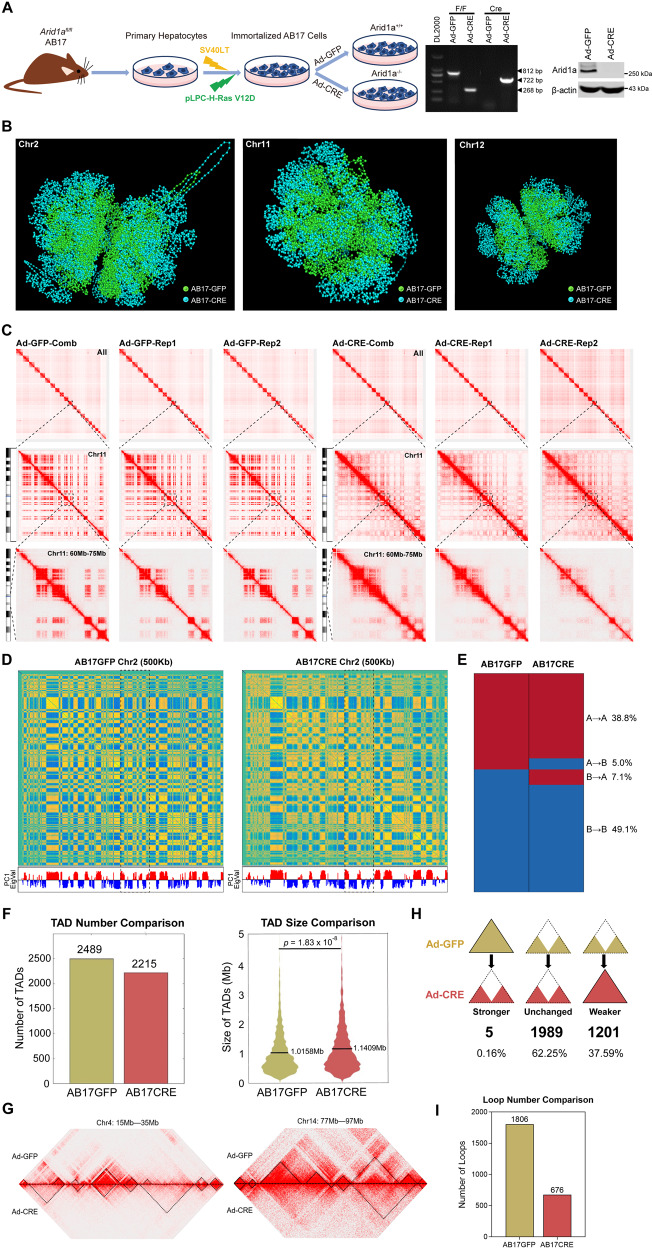
*Arid1a* deficiency alters chromatin conformation. **A** Schematic flowchart of the immortalized primary hepatocytes termed AB17, isolated from the mouse with *Arid1a*^*fl/fl*^ genotype and infected by SV40LT and pLPC-H-Ras V12D viruses successively (Left). PCR and Western blotting to identify the knockout effect of AB17 cells post Ad-GFP or Ad-CRE infection (Right). **B** The comparison on chromatin 3D simulation of Chromosome 2 (Chr2), Chr11, and Chr12 between *Arid1a* WT (Ad-GFP) and KO (Ad-Cre) AB17 cells. **C** All chromatins at 1-Mb resolution (Top), a zoom-in of Chr11 at 250-kb resolution (Middle) and Chr11: 60-75 Mb at 25-kb resolution (Bottom) of the replicates and combined contact metrics in *Arid1a* WT (Ad-GFP) and KO (Ad-Cre) AB17 cells. **D** Hi-C contact maps of Chr2 in *Arid1a* WT (Ad-GFP) and KO (Ad-Cre) AB17 cells at 500-kb resolution (Top). Compartment A (red) and B (blue) are shown by PC1 eigenvectors (Bottom). Dashed boxes indicate the representative compartment switch in Chr2. **E** Percentages of compartment switching between *Arid1a* WT (Ad-GFP) and KO (Ad-Cre) AB17 cells. **F** The comparison on TAD number (Left) and average size in Mb (Right) between *Arid1a* WT (Ad-GFP) and KO (Ad-Cre) cells at 50-kb resolution. **G** Examples of TAD (encircled by dotted triangles) alteration in number and size. Shown in Hi-C contact maps, the larger domains were often observed in *Arid1a* KO (Ad-Cre) cells (Left, 15-35 Mb in Chr4); TADs were merged or vanished causing the visible reduction in number accompanied by the significantly larger sizes in *Arid1a* KO (Ad-Cre) cells (Right, 77-97 Mb in Chr14). **H** The statistics of changes in border strength induced by *Arid1a* deficiency from three categories: stronger, unchanged, and weaker. **I** The comparison on chromatin loop number between *Arid1a* WT and KO cells at 25-kb resolution.

To address the effect of Arid1a on chromatin conformation in hepatocytes, we first established *Arid1a* KO and WT AB17 hepatocytes (Fig. 1A). Then in situ DNase Hi-C assay was employed to generate chromatin interaction heatmaps by combining two pairs biological replicates (Figure S1A). Based on whole-genome and intra-chromosomal Hi-C data, we performed computational chromatin 3D simulation, indicating that the chromatin interaction was loosened upon *Arid1a* deficiency (Fig. 1B). Besides, the “checkerboard” pattern of contact matrices was visibly weakened in the *Arid1a* KO cells (Fig. 1C, D).

We further analyzed the conformational changes from three folding scales: compartments, TADs, and chromatin loops. Correlation and PCA computing on 500-kb binned Hi-C heatmaps were adopted to partition the whole genome into two eigenvalue groups: active histone marks H3K27ac and H3K4me1 enriched in compartment A as well as repressive markers H3K27me3 and H3K9me3 aggregated in compartment B (Figure S1B). Through comparison of the compartments identified in *Arid1a* WT and KO AB17 cells (Fig. 1D and S1C), the results showed that approximately 12.1% compartments flipped upon *Arid1a* depletion (Fig. 1E; Tables S3.1 and S3.2).

Next, we investigated the conformational changes at sub-megabase scale using our homemade TAD calling algorithm TopDom [24]. Through comparing TADs in the control and *Arid1a* KO cells (Tables S3.3 and S3.4), interestingly, we found that the number of TADs was reduced but their sizes were significantly larger in the AB17 hepatocytes without *Arid1a* (Fig. 1F, G), implying that TAD disruption and convergence may be relevant to changes on the insulation of boundaries induced by *Arid1a* deficiency. Through the calculation on the signals from inter-TAD genomic bins, the majority of affected boundaries were observed significantly weakened as *Arid1a* loss (Fig. 1H), supporting that *Arid1a* depletion mediates the boundary strength changes and TAD remodeling. To examine whether a compartment switch was impacted by boundary strength in a likewise manner, we analyzed the changes of bin signal tendency in insulation boundaries during compartment switch. The result showed that stable compartments exhibited noticeable changes while virtually slight undulations were detected in switched compartments (Figure S1D). Additionally, in both boundary-weakened and -stronger TADs, the stable compartments account for the majority (Figure S1E), indicating that the *Arid1a* deficiency-induced boundary strength weakening cannot lead to compartment switch at megabase scale.

We finally explored the conformational changes on chromatin loops at 25-kb resolution (Tables S3.5 and S3.6). The number of loop domains was significantly decreased upon *Arid1a* depletion (Fig. 1I), suggesting the essential role of Arid1a in chromatin loop maintenance.

Taken together, these findings indicate that *Arid1a* deficiency alters chromatin conformation reflected in compartment switching, TAD remodeling, and chromatin loop weakening.

### SWI/SNF complex associates with chromatin architecture via BRG1-RAD21 axis

**Fig. 2:**
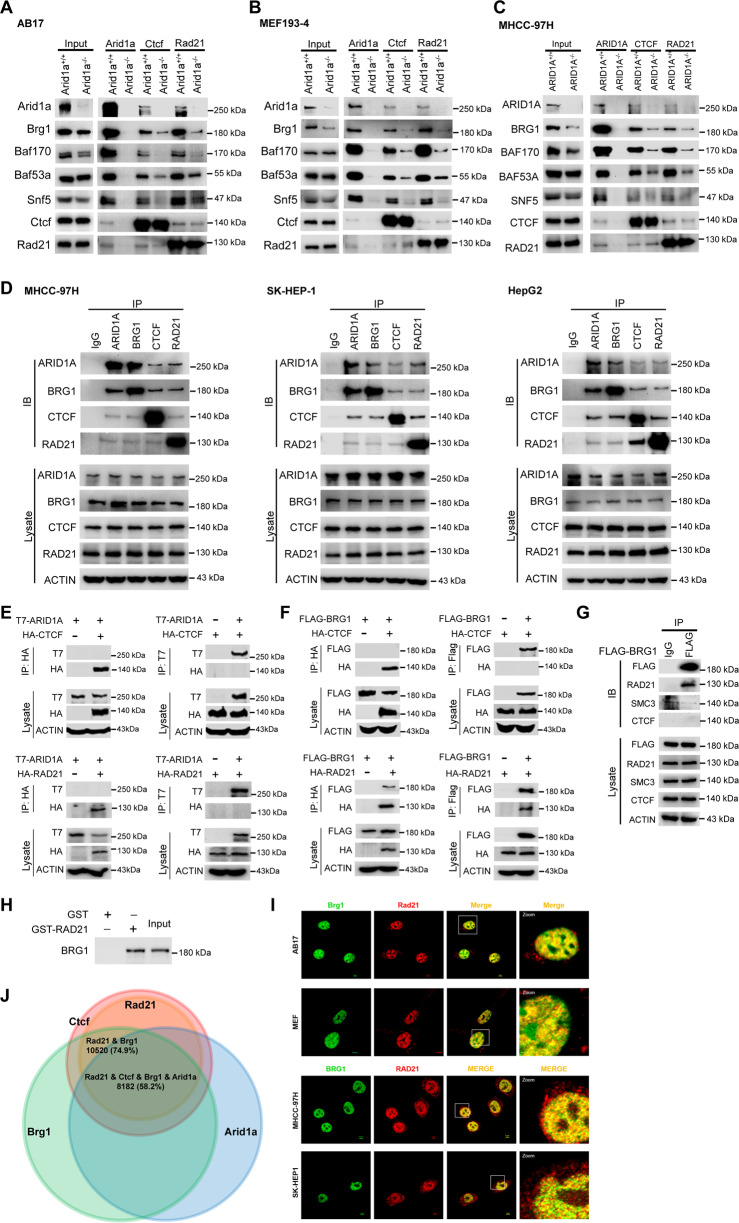
SWI/SNF complex associates with chromatin architecture via BRG1-RAD21 axis. **A-C**. Endogenous co-IP assay with anti-ARID1A, CTCF, and RAD21 antibodies was performed in *ARID1A* WT and KO AB17 **A**, MEF cells (193-4), **B** and human HCC MHCC-97H cells **C**, and then blotting assay detected with the indicated antibodies. **D** Validation for the interaction between ARID1A/BRG1 and architectural elements CTCF/RAD21 in human liver cancer cell lines MHCC-97H, SK-HEP-1 and HepG2. **E** HEK293T cells transfected with the indicated constructs were subjected to co-IP assay with anti-T7 and -HA antibodies to verify the interaction between ARID1A and CTCF (Top) or RAD21 (Bottom). **F** The same as **E** but for detecting the interaction between BRG1 and CTCF (Top) or RAD21 (Bottom) with anti-Flag and -HA antibodies. **G** Validation for the interaction between BRG1 and RAD21 by co-IP assay in Flag-tagged BRG1-transfected HEK293T cells. **H** Glutathione S-transferase (GST) pull-down experiments using GST-RAD21 protein and in vitro translated BRG1 protein. BRG1 protein binding to GST or GST-RAD21 was detected by the anti-BRG1 antibody. **I** Immunofluorescent staining showing the colocalization of both BRG1 (green) and RAD21 (red) in mouse AB17, MEF cells (193-4), as well as human liver cancer MHCC-97H and SK-HEP-1 cells (Scale bar, 5 µm). **J** The ChIP-seq peaks of Ctcf, Rad21, Arid1a, and Brg1 were extensively overlapped in the genome distribution.

As known, TADs and loops are established and maintained by the architectural elements, CTCF, and cohesin complex [37]. To determine the underlying molecular basis by which ARID1A affects the chromatin organization, we performed co-IP assays in *ARID1A* WT and KO AB17 (Fig. 2A), MEFs (Fig. 2B), and MHCC-97H cells (Fig. 2C), respectively. The results demonstrated that, besides ARID1A, both CTCF and RAD21 as baits also pulled down some core SWI/SNF complex subunits. Interestingly, in the absence of *ARID1A*, the associations between SWI/SNF complex subunits and CTCF/RAD21 were significantly weakened (Fig. 2A-C), implying that the role of SWI/SNF complex in maintaining chromatin organization could be impaired once *ARID1A* was deficient.

In light of BRG1 as the core ATPase of SWI/SNF complex and the most obviously inhibited subunit upon *ARID1A* depletion (input lanes of Fig. 2A-C), next we focused to reveal the relationship among ARID1A, BRG1, and the architectural elements CTCF and RAD21. The association between ARID1A/BRG1 and CTCF/RAD21 was affirmed in human liver cancer cell lines in vivo (Fig. 2D). To examine whether ARID1A or BRG1 can directly interact with CTCF or RAD21, we carried out exogenous IP assays with different tags. Except for the binding of BRG1 to RAD21, no direct interaction existed among other proteins (Fig. 2E, F). Additionally, BRG1 specifically interacted with RAD21 but not SMC3, the skeleton protein of cohesin (Fig. 2G). The transcribed/translated BRG1 could be pulled down by the GST-RAD21 protein but not the GST alone, further confirming that BRG1 could directly bind to RAD21 in vitro (Fig. 2H).

Furthermore, BRG1 partially co-localized with RAD21 in the nucleus (Fig. 2I). And the genome distribution of Brg1, Ctcf, and Rad21 were extensively overlapped (Fig. 2J), implying that SWI/SNF complex might be a cooperator for architectural elements in chromatin organization. To sum up, these findings reveal that SWI/SNF complex associates with CTCF and cohesin via the direct physical interaction between BRG1 and RAD21.

### *ARID1A* deficiency weakens BRG1-RAD21 axis via BRG1 suppression

**Fig. 3:**
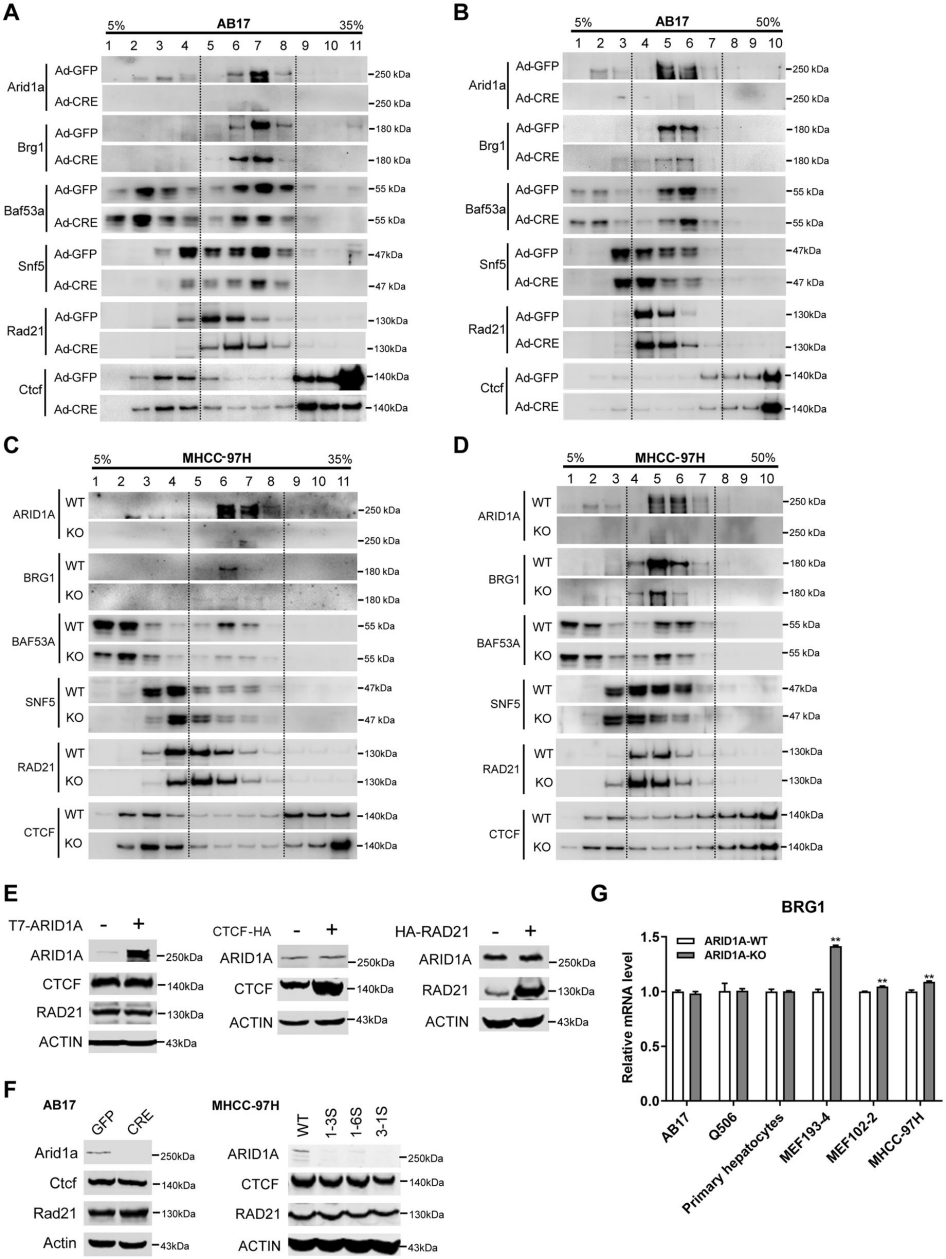
*ARID1A* deficiency weakens BRG1-RAD21 axis via BRG1 suppression. **A** Glycerol sedimentation assay with 5 to 35% gradients for detecting the co-segregation of Brg1 and Rad21 in nuclear fractions in *Arid1a* WT and KO AB17 cells. The dotted lines enclose the co-density stratifications of Brg1 and Rad21. **B** Sucrose sedimentation (5 to 50%) assay in *Arid1a* WT and KO AB17 cells. The co-fractionation of Brg1 and Rad21 circled with dotted lines. **C**, **D** The same as **A** and **B** respectively but in HCC cell line MHCC-97H. **E** HEK293T cells transfected with the T7-ARID1A construct exhibited no significant impact on CTCF and RAD21 (Left). Similarly, no impact on ARID1A in CTCF (Medium) and RAD21 over-expressed cells (Right). **F** Neither CTCF nor RAD21 was effected by *ARID1A* deficiency in AB17 (Left) and MHCC-97H cells (Right). **G** Brg1 mRNA expression levels in *Arid1a* WT and KO AB17 cells, Q506 cells, primary hepatocytes, MEFs (193-4 and 102-2 cells), and HCC cell line MHCC-97H with three biological replicates. Error bars were painted by SEM. *p* value was calculated by a two-tailed Student’s *t*-test. mRNA expression of *Gapdh* was employed as an internal control.

We carried out glycerol and sucrose gradient sedimentation assays to examine whether *ARID1A* deficiency attenuated the connection between BRG1 and RAD21. The results showed that the amount of Brg1 was obviously decreased, while Brg1 and Rad21 still maintained in the same density stratification in *Arid1a*^–/–^ AB17 cells (Fig. 3A, B). Similarly, the co-segregation of BRG1 and RAD21 was not affected by *ARID1A* deficiency in MHCC-97H cells (Fig. 3C, D). These data indicated that the deficiency of *ARID1A* cannot disrupt the co-segregation of BRG1 and RAD21. Moreover, we also excluded the mutual regulatory effects between ARID1A and CTCF/RAD21 (Fig. 3E, F).

As input lanes are shown in Fig. 2A-C and Fig. 3A-D, the expression of BRG1 was mightily suppressed in *ARID1A*^−/−^ cells, which could be responsible for the weakened interaction between BRG1 and RAD21. Then, we examined the mRNA expression changes of Brg1. Except for the Brg1 mRNA showing no significant difference between *Arid1a* WT and KO AB17 cells, we also did not observe the downregulation of Brg1 in *Arid1a-*depleted Q506, primary hepatocytes, MEFs, and HCC cell line MHCC-97H (Fig. 3G). Therefore, it can be conjectured that the suppressed BRG1 in ARID1A KO cells could not be the regulatory outcome at the transcriptional level, but possibly due to the post-translational effect such as accelerated protein degradation, which is worthy of further investigation.

Taken together, *ARID1A* deficiency cannot block the interaction between BRG1 and RAD21, or impact the expression of CTCF and RAD21. It is more reasonable that insufficient BRG1 triggered by *ARID1A* depletion results in weakened interaction.

### Compartment switching dysregulates cancer-related genes

**Fig. 4:**
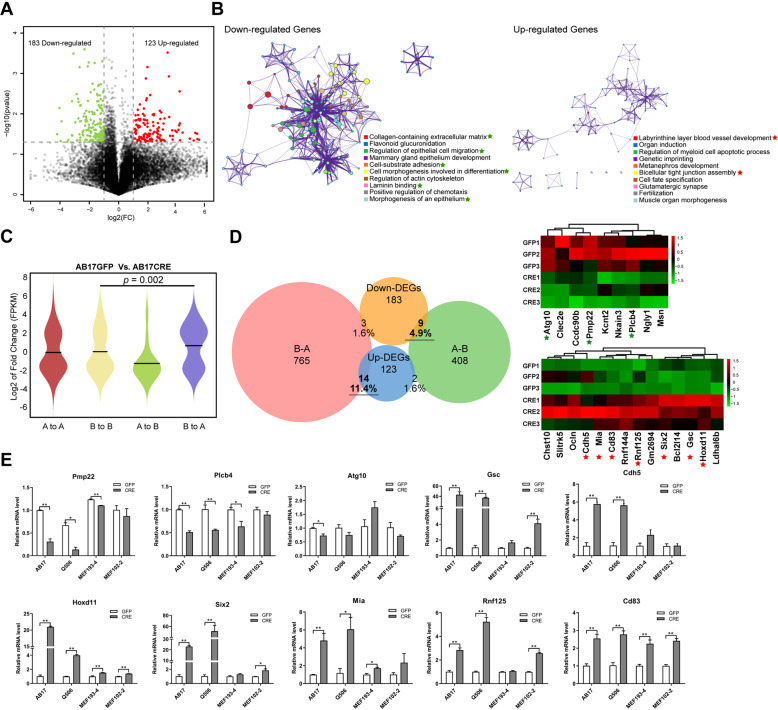
Compartment switching dysregulates cancer-related genes. **A** Volcano plot showing the DEGs (fold change >2, *p* value <0.05) between *Arid1a* WT (Ad-GFP) and KO (Ad-Cre) AB17 cells. The green and red dots represent down- and up-regulated genes post *Arid1a* deficiency, respectively. **B** The enrichment analysis on down- (Left) and up-regulated (Right) genes. The top ten significantly enriched terms were shown (*p* value <0.01). The color-coded circles symbolize the enriched GO terms and pathways. **C** Fold change (Ad-Cre/Ad-GFP log2) of mRNA expression (FPKM) of the genes residing at regions with compartment switching. **D** Venn diagrams displaying the overlap of DEGs and A/B compartment switch-related genes (Left). The heatmap showing the expression profiles of the switching compartments controlled-genes with triplicate datasets, of which down- and up-regulated cancer-related genes labelled by green and red pentagrams, respectively (Right). **E** qRT-PCR for the compartment switching-controlled genes in *Arid1a* WT (Ad-GFP) and KO (Ad-Cre) AB17 and Q506 hepatocytes as well as MEFs (193-4 and 102-2 cells) with three biological replicates. Error bars were painted by SEM. *p* value was calculated by a two-tailed Student’s *t*-test. mRNA expression of *Gapdh* was employed as an internal control.

To determine the biological processes effected by the perturbed conformation, we first obtained transcriptomic profiles of hepatocytes. As shown in Fig. 4A, there were 183 down-regulated genes and 123 up-regulated genes upon *Arid1a* depletion. Enrichment analysis showed that DEGs were significantly enriched in cell morphology-related terms, especially including extracellular matrix (ECM), cell migration, cell adhesion, and cell junction (Fig. 4B, Tables S4.1 and S4.2). Moreover, we performed Reactome analysis on DEGs, the results showed that, as *Arid1a* depletion, an extensive effect exerted on biological processes involving the previously reported pathways including packing of telomere ends and a series of cancer-related processes (Figures S2A, B).

Considering that active and inactive chromatins spatially in the nucleus are reflected by the formation of compartments [8, 38], we determined to explore *Arid1a*-deficiency mediated biological processes from the perspective of compartment switching. As shown in Fig. 4C, the switched compartments were visibly correlated with gene expression changes as *Arid1a* loss, suggesting that Arid1a could serve as an underlying modulator at the megabase scale. Furthermore, 23 of DEGs modulated by the A/B compartment switching were identified as the candidates (Fig. 4D), disregarding those in stable compartments (Table S4.3). Notably, through enrichment analyses (Tables S4.4 and S4.5) combined with the searching literatures manually, we observed that the majority of these genes have been reported to play vital roles in tumorigenesis. The qRT-PCR data verified in AB17, Q506, and MEF cells were in accordance with RNA-seq datasets (Fig. 4E).

Collectively, these findings manifest that compartment switching induced by *Arid1a* depletion modulates the transcription of some cancer-related genes, such as *Pmp22* [39, 40], *Atg10* [41], *Gsc* [42, 43], *Rnf125* [44], and *Cdh5* [45], which have been known to be involved in tumorigenesis.

### Weakened chromatin loops and remodeled TADs cause aberrant transcription

Through calling and comparing TADs and loop domains derived from Hi-C matrices, we summed up the regulatory pattern that silenced genes (A to B switching) were mainly due to the weakened enhancer-promoter (E-P) loops and the blocked enhancers owing to new-generated TAD boundaries; whereas activated genes (B to A switching) were mainly due to proximal *cis*-elements on account of TAD formation and repositioning (Figures S3A-C).

**Fig. 5:**
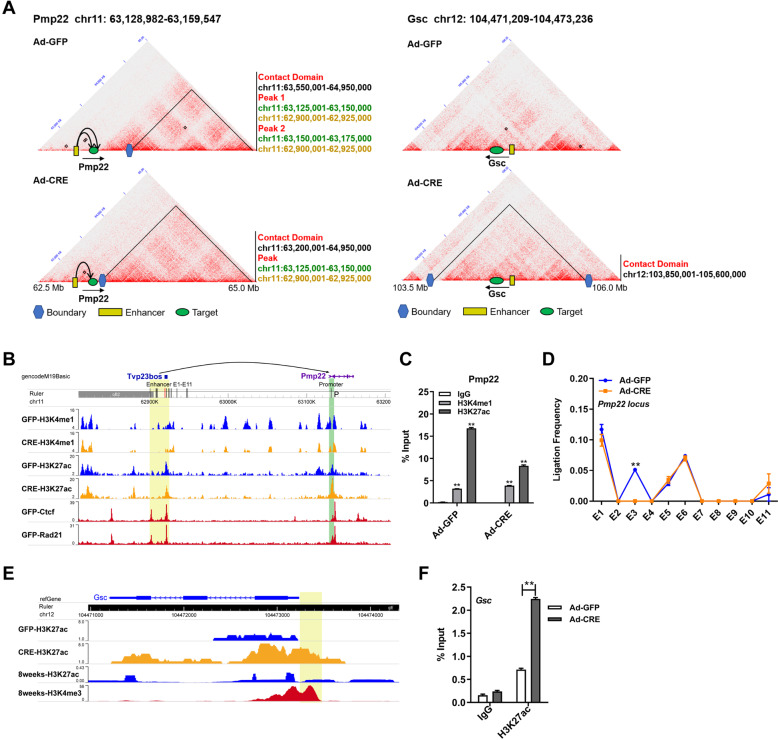
Weakened chromatin loops and remodeled TADs cause aberrant transcription. **A** Hi-C contact maps of *Pmp22* and *Gsc*, which were dysregulated in the switched compartments caused by *Arid1a* deficiency. TADs (Contact domains) are visible as triangle-shaped regions with a high frequency of interactions, and the position information are labelled in black font. Locally enriched peaks discovered via HiCCUPS are referred to as chromatin loops, and location coordinates are shown in green (the target gene) and yellow (the potential enhancers) font in line with the colors of graphic symbols. **B** Expanded views of the potential enhancer locus and proximal promoter of *Pmp22*, with H3K4me1 and H3K27ac ChIP-seq tracks for recognition on the potential enhancers, and Ctcf and Rad21 ChIP-seq tracks for pointing the anchored loops in AB17 cells. Vertical black bars label the enhancers and promoter primers examined in the 3C assay. **C** Identification on enhancers of E-P loops in *Pmp22*. The position of primer for the enhancer is labelled by the vertical red bar. Histone marks H3K27ac and H3K4me1 were significantly enriched at enhancer locus of *Pmp22* in AB17 with IgG as control. **D** Curve graphs indicate the significantly reduced ligation frequencies at E-P anchored loops of *Pmp22* in *Arid1a*-deficient cells. **E** Validation on a regulatory pattern of the up-regulated gene *Gsc* within switched A compartment. H3K27ac and H3K4me3 ChIP-seq tracks were obtained from AB17 cells and ENCODE database (H3K27ac: ENCSR000CDH; H3K4me3: ENCSR000CAP), and the location of enhancer boxed with the yellow rectangle. **F** A significantly increased the H3K27ac signal of the *Gsc* gene detected in *Arid1a* knockout cells with IgG as control.

Next, we investigated some representative genes from the dys-regulatory patterns: loop decreased (*Pmp22*) and TAD remodeling (*Gsc*) (Fig. 5A), to verify the above-mentioned tentative pattern mediated by *Arid1a* loss. Based on the enhancers identified by the peak signal of H3K4me1 and H3K27ac (Fig. 5B, C), 3C assays were performed on *Pmp22*, showing that the ligation frequencies of E-P loops at *Pmp22* locus were decreased in *Arid1a* KO AB17 cells, which precisely agreed with the above pattern (Fig. 5D). We further estimated the interaction peaks in *PMP22* locus combined Hi-C matrices of HepG2 and GM12878 cells as referred (Figure S4A). Through the ChIP-seq tracks in HepG2 cells, we defined the enhancer of the *PMP22* gene (Figures S4B, C). Additionally, similar alterations in *PMP22* were also observed in human MHCC-97H cells (Figure S4D).

For B-A transition gene *Gsc*, the distinct peaks of H3K27ac were observed in *Arid1a*-KO AB17 cells but not in *Arid1a*-WT ones (Fig. 5E). ChIP-qPCR analysis revealed that H3K27ac were significantly enriched at the upstream of *Gsc* upon *Arid1a* deficiency (Fig. 5F), indicating that *Gsc* was activated through the proximal *cis*-element. And similar results were observed in MHCC-97H cells (Figures S4E, F). Furthermore, the regulatory patterns were further confirmed in another liver cancer cell line SK-HEP-1 (Figures S4G, H). Additionally, we performed 3C and ChIP-qPCR assays in human endometrial cancer cell lines ISK and HEC-1-A, because *ARID1A* mutations are prevalent in this type of cancer. Interestingly, *ARID1A* knockdown has no significant effect on the E-P contact of *PMP22*, H3K27ac signal at the upstream of *GSC*, and their expression levels of *PMP22* and *GSC* in ISK and HEC-1-A cells (Figures S4I-N), suggesting that the role of ARID1A in higher-order genome organization might depend on cell types. However, the existed loss-of-function *ARID1A* mutations in these endometrial cancer cells could have disrupted the chromatin conformational regulation on *PMP22* and *GSC* loci.

To confirm the effect of Arid1a on chromatin organization, we designed the rescue experiment. The contact frequencies of E-P loops in *PMP22* as well as H3K27ac enrichment levels at the enhancer of *GSC* could be restored by ectopic ARID1A to a significant extent in both AB17 and MHCC-97H cells (Figure S5).

Overall, these data revealed that the weakened long-range chromatin loops and remodeled TADs were the underlying regulatory mechanism of aberrant gene expression induced by *Arid1a* deficiency.

### *ARID1A* deficiency promotes liver cancer cell metastasis via dysregulation of certain genes

**Fig. 6:**
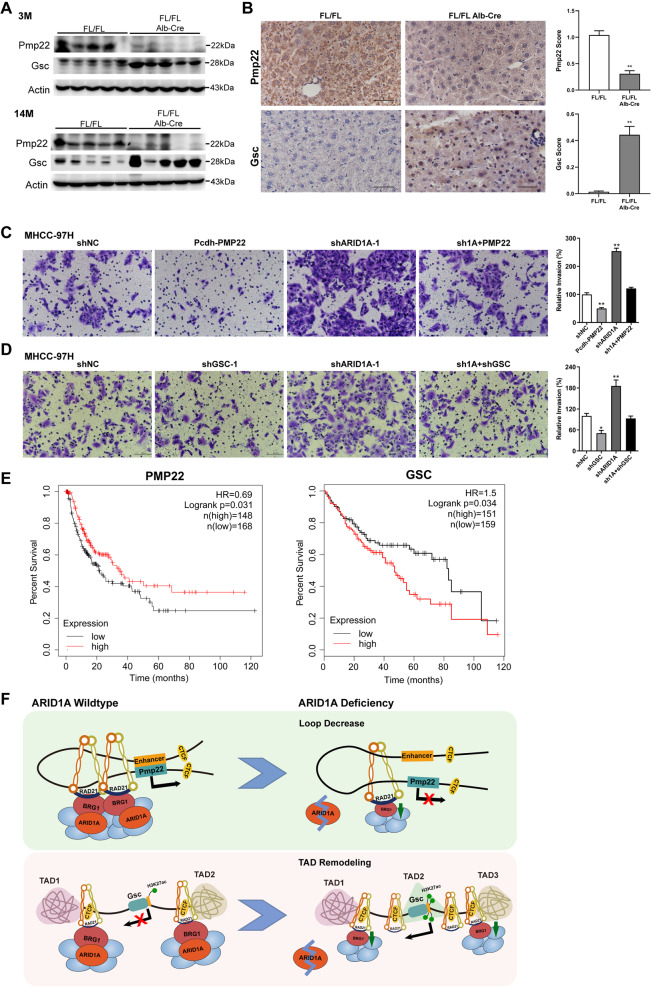
*ARID1A* deficiency promotes liver cancer cell metastasis via dysregulation on *PMP22* and *GSC*. **A** Immunoblotting on Pmp22 and Gsc in liver tissues of 3 and 14 month old *Arid1a*^*fl/fl*^ mice and *Arid1a*^*LKO*^ littermates. **B** Representative IHC staining (Left) and quantification of positive staining (Right) for Pmp22 and Gsc in liver tissues of 3-month-old *Arid1a*^*fl/fl*^ mice and *Arid1a*^*LKO*^ littermates. Scale bar = 50 μm. Data are presented as the mean ± SEM (*n* = 5 per group). *p < 0.05, **p < 0.01, calculated by a two-tailed Student’s *t*-test. **C** Representative images of invasive MHCC-97H cells transfected with Pcdh-PMP22 construct; lentiviral shARID1A-1, and *ARID1A*-knockdown cells co-transfected with Pcdh-PMP22 in transwell assays (Scale bar, 100 µm) (Left) and quantification of the invasive cells (Right). Data are shown as mean ± SEM of three independent experiments. *p < 0.05, **p < 0.01, calculated by a two-tailed Student’s *t*-test. **D** Representative images and quantification of the invasive MHCC-97H cells treated with the indicated lentiviral shRNA plasmids for *ARID1A* (shARID1A-1) and *GSC* (shGSC-1) in transwell assays. **E** The relapse-free survival and the overall survival in HCC patients with high or low expression of *PMP22* (Left) and *GSC* (Right), examined by Kaplan-Meier analysis. **F** Schematic diagram of the dysregulated mechanism related to *PMP22* and *GSC* loci upon *ARID1A* deficiency. Based on our data, we proposed the working model of the SWI/SNF complex in maintaining chromatin organization mediated by the BRG1-RAD21 axis in hepatocytes. Once *ARID1A* is deficient, the chromatin loop tethering promoter to the enhancer of *Pmp22* might be weakened due to the attenuated anchorage force arising from insufficient BRG1 (Top); the unaided boundaries lead to TAD remodeling surrounding *Gsc* locus to some extent (Bottom).

Next we investigated the expression levels of these aberrant genes in vivo. The results showed that Pmp22 was markedly repressed, whereas Gsc was significantly elevated in liver tissues of *Arid1a*^*LKO*^ and *Ubc-CreER*^*T2*^; *Arid1a*^*fl/fl*^ mice, in comparison with that from the *Arid1a* WT littermates (Fig. 6A and S6A). Gel source images were available in Figure S7. Meanwhile, IHC staining results further verified the expression changes (Fig. 6B and S6B). These data affirmed that *Arid1a* deficiency leads to the similar dysregulation of these genes in vivo.

It has been known that *ARID1A* deficiency promotes liver cancer metastasis, therefore, whether these ARID1A-regulated genes can contribute to the malignant behaviors need to be confirmed. The expression efficiency of ectopic *PMP22* as well as the silencing effects targeting *ARID1A* and *GSC* were evaluated in MHCC-97H and SK-HEP-1 cells (Figure S6C). Here the enforced *PMP22* overexpression significantly inhibited the invasion capacity (Fig. 6C and S6D), in line with the feature of tumor suppressor. In accord with the previous study [42, 43], *GSC* knockdown significantly repressed the invasion capacity in liver cancer cells (Fig. 6D and S6E). These data suggested that PMP22 may act as a potential suppressor in liver cancer metastasis, whereas GSC can promote cell invasion.

Then we further examined whether the dysregulated genes play crucial roles in *ARID1A* deficiency-driven liver cancer cell invasion. As expectedly, knockdown of *ARID1A* enhanced the invasive capacity, however, it was visibly inverted by the overexpressing *PMP22* simultaneously (Fig. 6C and S6D). Conversely, the increased invasion triggered by *ARID1A* knockdown can be abolished via silencing *GSC* expression (Fig. 6D and S6E). Additionally, the invasion capacity of these dysregulated genes *PMP22* and *GSC* was further confirmed with the other independent sets of shRNAs in MHCC-97H (Figures S6F, G) and SK-HEP-1 cells (Figures S6H, I). Moreover, high expression of *PMP22* negatively correlates with a decrease in relapse-free survival across HCC patients significantly, while conversely in *GSC* expression, indicating that the aberrant *PMP22* and *GSC*-gene signatures are the important events in HCC progression (Fig. 6E). These findings indicated that *ARID1A* deficiency-driven liver cancer metastasis might depend upon chromatin conformational dysregulation on some key genes like *PMP22* and *GSC* (Fig. 6F).

## Discussion

It has been elucidated that the chromatin accessibility alters followed by phenotypic abnormality once the deficient expression of SWI/SNF components occurs, e.g., *ARID1A* in HCT116 colorectal cancer cells and injury-induced liver-progenitor-like cells [46, 47]. However, except for the canonical character in accessibility, there is currently no definite role of SWI/SNF complex in chromatin architecture, especially in hepatocytes.

SWI/SNF complex is important for chromatin organization, whereas the specific roles depending on the context [19, 20]. Notably, our study for the first time deciphered the role of Arid1a in chromatin conformation of hepatocytes, which modulates compartment switching, TAD remodeling as well as sustain E-P looping (Fig. 1). It is the opposite character of Arid1a in TAD border strength, that we observed a decreased number of TADs in *Arid1a*-deficient hepatocytes, while an increased number of TADs called in OCCC cells [20].

Recent studies have signified the potential interaction between SWI/SNF complex and architectural elements, for instance, the extensive overlap on binding sites of SWI/SNF components, Pol II and CTCF in a genome-wide scale [48], and the functional cooperativity among CTCF, BRG1 and topoisomerase II beta (TOP2B) in the organization of TADs [49]. Marino et al. experimentally verify the reciprocity of CTCF with BRG1 and ARID1A in WiT49 cell line [50]. Furthermore, SWI/SNF complex is possibly associated with condensin II in OCCC cell lines [20]. Significantly, in this study, we elucidated the BRG1-RAD21 axis as the molecular basis of the SWI/SNF complex in the 3D genome (Fig. 2). The evidences that RAD21 directly interplays with BRG1 but not ARID1A, exactly agree with the latest proposed cryo-EM structure of human BAF bound to the nucleosome, of which ARID1A acts as the rigid core of Base modules to support the ATPase motor BRG1 contiguously positioning on nucleosome [51].

To explain the specific effect of ARID1A on the BRG1-RAD21 axis, we propose two putative models whereby *ARID1A* deficiency leads to the dissociation of SWI/SNF complex, or the suppression of BRG1/RAD21. For the first inference, a prior report has clarified ARID1A to be critical for the assembly of the ATPase module [52]. Recently, Wang et al. reported that both *ARID1s* (*ARID1A* and *ARID1B*) loss led to the splitting of cBAF into subcomplexes, involving the principal BRG1-containing complex and residual fractions [53]. Noteworthily, most of the other subunits are retained in the BRG1-containing subcomplex in contrast of the above-mentioned cBAF assembly hypothesis in which BRG1 recruitment depends on ARID1 proteins [52, 53]. Consistently, we observed that *ARID1A* depletion cannot destroy the distribution of subunits, or block the interaction between BRG1 and RAD21 (Fig. 3A-D). Furthermore, the results showed that BRG1 protein was significantly inhibited upon *ARID1A* loss, suggesting that ARID1A might participate in the BRG1-RAD21 axis through regulating BRG1 expression at the post-translational level (Figs. 2A-C and 3). Similarly, *ARID1A* deficiency-mediated degradation of the components of SWI/SNF complex have been observed, such as DPF2 in *ARID1*-less H2.35 hepatocyte cell line, BAF170 in *ARID1s*-knockout livers [53], as well as BRG1, BAF155, BAF170, and SNF5 in *ARID1A*-depleted MEF cells [54].

Previous studies indicated that defective *ARID1A* can explicitly induce cell plasticity such as the acquisition of stem cell-like traits and invasion properties in hepatocytes [55, 56], endometrial epithelial cells [57], and pancreatic ductal cells [58], which are resorted to YAP/TAZ response or EMT-related signaling pathways. This study aims to reveal the molecular mechanism of liver cancer metastasis driven by *ARID1A* deficiency from chromatin conformation perspective. Except for PMP22 and GSC mentioned above, the downregulation of ATG10, with A to B transition, may facilitate metastasis of colorectal cancer cells [41]; transcription factor SIX2, with B to A switch, has been reported as a critical regulator for the stemness of breast cancer cells that enables metastatic colonization [59, 60]. These findings revealed that *ARID1A* deficiency-induced conformational dysregulation may trigger or suppress some key metastasis-related genes that contribute to liver cancer progression. Noteworthily, cohesin is reported responsible for EMT/MET via chromatin interactions of mesenchymal genes *TGFB1* and *ITGA5* loci [61], implying the significance of conformational changes in cancer metastasis process.

This study reveals the crucial role of the SWI/SNF complex in maintaining chromatin organization in hepatocytes, which is mediated by the core subunit ATPase BRG1 interacting with the architectural element RAD21. Interestingly, *ARID1A* deficiency attenuates the BRG1-RAD21 axis via suppressing the BRG1 expression level, which is insufficient to facilitate RAD21 keeping higher-order chromatin structure, resulting in TADs remodeling and deterrent chromatin loops. Notably, the abnormally expressed genes caused by the conformational changes due to *Arid1a* deficiency are significantly enriched in cancer-related processes, some of which are further verified to be involved in *ARID1A* deficiency-driven liver cancer cell invasion. This study reveals a novel function of SWI/SNF complex on chromatin conformation apart from the canonical nucleosome remodeling in hepatocytes, and also provides new insights into liver cancer tumorigenesis and progression.

## Supplementary information

Supplementary Figure legends

Figure S1

Figure S2

Figure S3

Figure S4

Figure S5

Figure S6

Figure S7

Table S1

Table S2

Table S3

Table S4

## Data Availability

High-throughput sequencing datasets (RNA-seq and ChIP-seq) have been deposited to NCBI Gene Expression Omnibus (GEO) database with the accession number GSE152052. Hi-C data are available from the Sequence Read Archive (SRA) under the accession number PRJNA637836.
