# Supplementary material for: ARID1A deficiency weakens BRG1-RAD21 interaction that jeopardizes chromatin compactness and drives liver cancer cell metastasis
Source: Cell Death Dis. 2021 Oct 23;12(11):990. doi: 10.1038/s41419-021-04291-6 (PMC8542038; doi:10.1038/s41419-021-04291-6)
Supplement: Supplementary file 1 — Supplementary Figure legends [file 41419_2021_4291_MOESM1_ESM.docx]

**Figure S1. Correlation analysis, peak signal of histone marks and border strength across compartment A/B.**

**A.** Spearman correlation coefficients from 1 Mb binned Hi-C *cis* contact matrices among replicates and combined ones. **B.** ChIP-seq signal density of the active histone marks H3K27ac and H3K4me1, and repressive marks H3K27me3 and H3K9me3 across compartments A and B. **C.** The comparison on compartments A/B in Chromosomes 2, 11 and 12 respectively between *Arid1a* WT and KO AB17 cells. Double-sided arrows labeling the representative compartment switch against *Arid1a* deficiency. **D.** The bin signal tendency around borders of stable (A to A and B to B), activated (B to A) and silent (A to B) compartments against *Arid1a* knockout. **E.** The statistics of border-changed TADs with A/B compartment switch: for the conserved TADs in *Arid1a* WT and KO cells.

**Figure S2.** **Pathway enrichment analysis on DEGs via Reactome Database.**

The significant enrichment pathways labelled in colored font (*p* < 0.05), of which the significant metastasis-related pathways shown in red font.

**Figure S3. Interaction heatmaps of aberrant cancer-related genes.**

**A.** Cartoon schematic depicts dysregulation patterns of the aberrant genes in switched A/B compartments including loop decrease, TAD formation and TAD repositioning. Graph symbols circled in the rectangle. **B.** Interaction heatmaps of the silenced genes in switched compartment B as *Arid1a* deficiency. The gene location circled by dotted lines. The intersected lines with the diagonal round into TADs. The quadrates label the positions of loop domains. **C.** Interaction heatmaps of up-regulated genes occurred B to A transition.

**Figure S4. Aberrant genes caused by weakened E-P loops and remodeled TADs in liver cancer cell lines.**

**A.** Heatmap depicts contact frequency between triangulated loci in HepG2 and GM12878 cells within a 700-Kb region containing *PMP22*, of which the contact domains are labelled by the horizontal colored stripes, and the E-P anchored loops in *PMP22* are marked by the dotted lines. The target gene *PMP22* and the potential enhancer are shown as green and yellow pentagrams, respectively. **B.** Expanded views of the ascertained enhancer locus and proximal promoter of *PMP22* shown with H3K27ac (ENCSR000AMO) and H3K4me1 (ENCSR000APV) ChIP-seq tracks as well as CTCF (ENCSR000AMA) and RAD21 (ENCSR054FKH) ChIP-seq tracks of HepG2 cells obtained from ENCODE database as refer. Vertical black bars label the tested primers’ location of enhancers and promoter in 3C assay. **C.** Identification on enhancers of E-P loops in *PMP22*. Histone marks H3K27ac and H3K4me1 were significantly enriched at enhancer locus of *PMP22* in MHCC-97H cells with IgG as control. **D.** Measurement on *PMP22* E-P contact frequency changes as *ARID1A* deficiency by 3C assay in MHCC-97H. Curve graph indicates the significantly reduced 3C ligation frequencies at the E-P anchored loop of *PMP22* in *ARID1A* deficient cells. **E.** Expanded views of the ascertained enhancer locus and proximal promoter of *PMP22* shown with H3K27ac (ENCSR000AMO), H3K4me1 (ENCSR000APV) and H3K4me3 (ENCSR575RRX) ChIP-seq tracks of HepG2 cells obtained from ENCODE database as refer. The enhancer boxed with a yellow rectangle. **F.** Validation for the up-regulated gene *GSC* controlled by B to A compartment switching. Significantly increased H3K27ac signals were detected in *ARID1A* KO cells, IgG served as control. **G.** Measurement on *PMP22* E-P contact frequency changes as *ARID1A* knockdown in SK-HEP-1 cells. **H.** Detection on H3K27ac peak signals of the dysregulated gene *GSC* induced by the silenced *ARID1A* in SK-HEP-1 cells. **I.** Detection on the silencing effects targeting *ARID1A* in endometrial cancer cell lines ISK and HEC-1-A. **J.** Measurement on *PMP22* E-P contact frequency changes as *ARID1A* knockdown in ISK cells. **K.** Detection on H3K27ac peak signals at the upstream of *GSC* against *ARID1A* deficiency in ISK cells. **L-M.** The same as (J-K), but in HEC-1-A cells. **N.** qRT-PCR for *PMP22* and *GSC* genes in endometrial cancer cell lines ISK and HEC-1-A against *ARID1A* knockdown. mRNA expression of *GAPDH* was employed as an internal control.

**Figure S5. Conformational changes of *Pmp22* and *Gsc* in *Arid1a*-depleted cells transfected with T7-ARID1A construct.**

**A.** The protein and mRNA levels of Arid1a restored after T7-ARID1A plasmid was transfected to *Arid1a*-depleted (Ad-Cre) AB17 cells. **B-C.** Detection on the ligation frequency at E-P anchored loops of *Pmp22* (**B**) and H3K27ac peak signal in *GSC* (**C**) in the *Arid1a*-deficient cells transfected with T7-ARID1A construct. **D-F.** The same as (**A-C**), but in MHCC-97H cells. The statistics on contact frequencies between *ARID1A* WT and KO cells labelled by * (**p* < 0.05, ** *p* < 0.01); the statistics between *ARID1A* KO and T7-ARID1A transfected KO cells labelled by # (#*p* < 0.05, ##*p* < 0.01); the statistics between *ARID1A* WT and T7-ARID1A transfected KO cells labelled by & (&*p* < 0.05, &&*p* < 0.01).

**Figure S6. *ARID1A* deficiency promotes liver cancer cell metastasis via dysregulation on *PMP22* and *GSC*.**

**A.** Validation for the expression of Pmp22 and Gsc in liver tissues of *Arid1a^fl/fl^* and *Ubc-CreER^T2^*; *Arid1a^fl/fl^* mice 1 and 3 months post tamoxifen induction. **B**. Representative IHC staining (Left) and quantification of positive staining (Right) for Pmp22 and Gsc in liver tissues of 3 month old *Arid1a^fl/fl^* and *Ubc-CreER^T2^*; *Arid1a^fl/fl^* mice. Scale bar = 50 μm. Data are presented as the mean ± SEM (n = 5 per group). **p* < 0.05, ***p* < 0.01, calculated by a two-tailed Student’s *t*-test. **C.** qRT-PCR was performed to evaluate the over-expression efficiency of ectopic *PMP22* and the silencing effects targeting *ARID1A* and *GSC* with two independent sets of shRNAs in MHCC-97H and SK-HEP-1 cells. **D.** Representative images of invasive SK-HEP-1 cells transfected with Pcdh-PMP22 construct; lentiviral shARID1A-1, and *ARID1A*-knockdown cells co-transfected with Pcdh-PMP22 in transwell assays (Scale bar, 100 µm) (Left) and quantification of the invasive cells (Right). Data shown as mean ± SEM of three independent experiments. **p* < 0.05, ***p* < 0.01, calculated by a two-tailed Student’s *t*-test. **E.** Representative images and quantification of the invasive SK-HEP-1 cells treated with the indicated lentiviral shRNA plasmids for *ARID1A* (shARID1A-1) and *GSC* (shGSC-1) in transwell assays. **F.** The effects of PMP22 on cell invasion were investigated by transwell assays in MHCC-97H transfected with Pcdh-PMP22 construct and the other independent set of shRNA targeting *ARID1A* (shARID1A-2). **G.** Representative images and quantification of the invasive MHCC-97H cells post treatment with independent sets of shRNAs for *ARID1A* (shARID1A-2) and *GSC* (shGSC-2). **H-I.** The same as (F-G), but in SK-HEP-1 cells.

**Figure S7. The whole untrimmed Western blotting images.**

**Table S1.1** Sequence information of shRNA, sgRNA and primers for the full length of PMP22

**Table S1.2** Oligonucleotides for Hi-C library

**Table S1.3** Primer sequences for quantitative Real-Time PCR

**Table S1.4** Primers for 3C and ChIP-qPCR assay

**Table S2** Information list of the datasets downloaded from ENCODE database

**Table S3.1** Genomic coordinates and information of A/B compartmentalization in Ad-GFP cells

**Table S3.2** Genomic coordinates and information of A/B compartmentalization in Ad-CRE cells

**Table S3.3** Genomic coordinates and information of TADs in Ad-GFP cells

**Table S3.4** Genomic coordinates and information of TADs in Ad-CRE cells

**Table S3.5** Genomic coordinates and information of loops in Ad-GFP cells

**Table S3.6** Genomic coordinates and information of loops in Ad-CRE cells

**Table S4.1** Enrichment analysis on the down-regulated genes upon *Arid1a* deficiency

**Table S4.2** Enrichment analysis on the up-regulated genes upon *Arid1a* deficiency

**Table S4.3** DEGs in A-A and B-B unchanged compartments

**Table S4.4** Enrichment analysis on the DEGs regulated by compartment switch via Metascape

**Table S4.5** Enrichment analysis on the DEGs regulated by compartment switch via Reactome database
