# Supplementary figures and images for: ARID1A deficiency weakens BRG1-RAD21 interaction that jeopardizes chromatin compactness and drives liver cancer cell metastasis
Source: Cell Death Dis. 2021 Oct 23;12(11):990. doi: 10.1038/s41419-021-04291-6 (PMC8542038; doi:10.1038/s41419-021-04291-6)

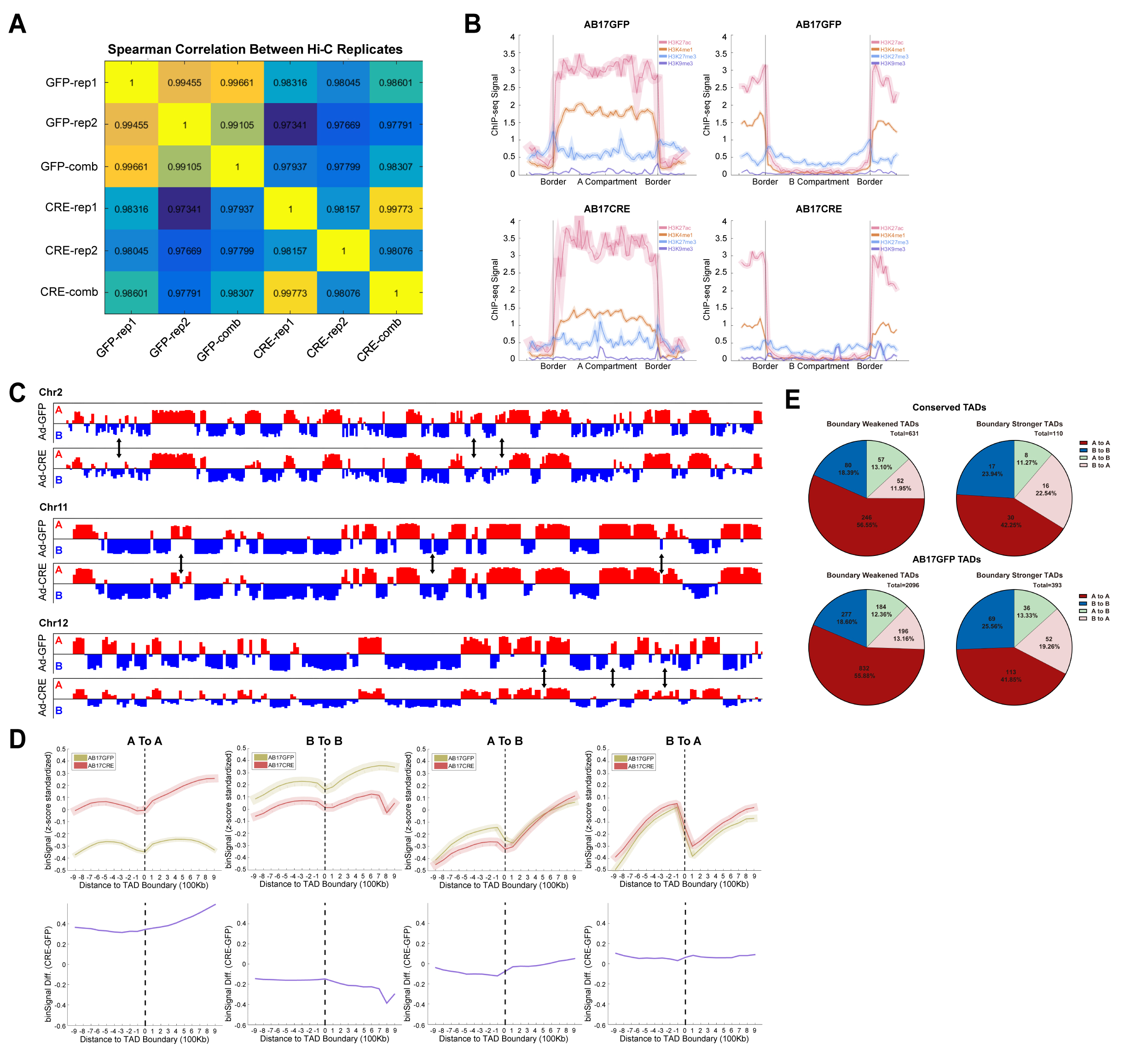

Supplement: Supplementary file 2 — Figure S1 [file 41419_2021_4291_MOESM2_ESM.jpg]

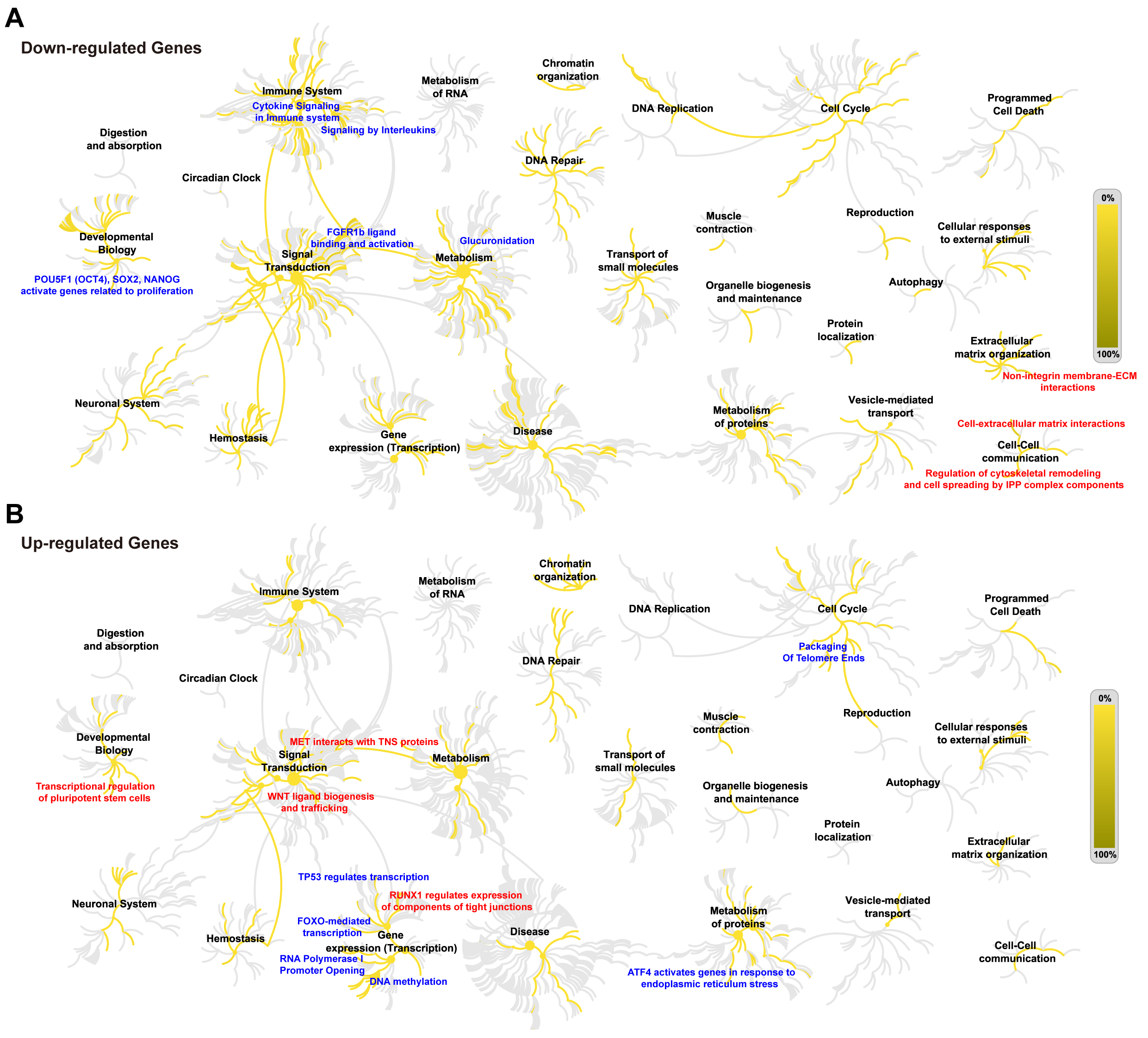

Supplement: Supplementary file 3 — Figure S2 [file 41419_2021_4291_MOESM3_ESM.jpg]

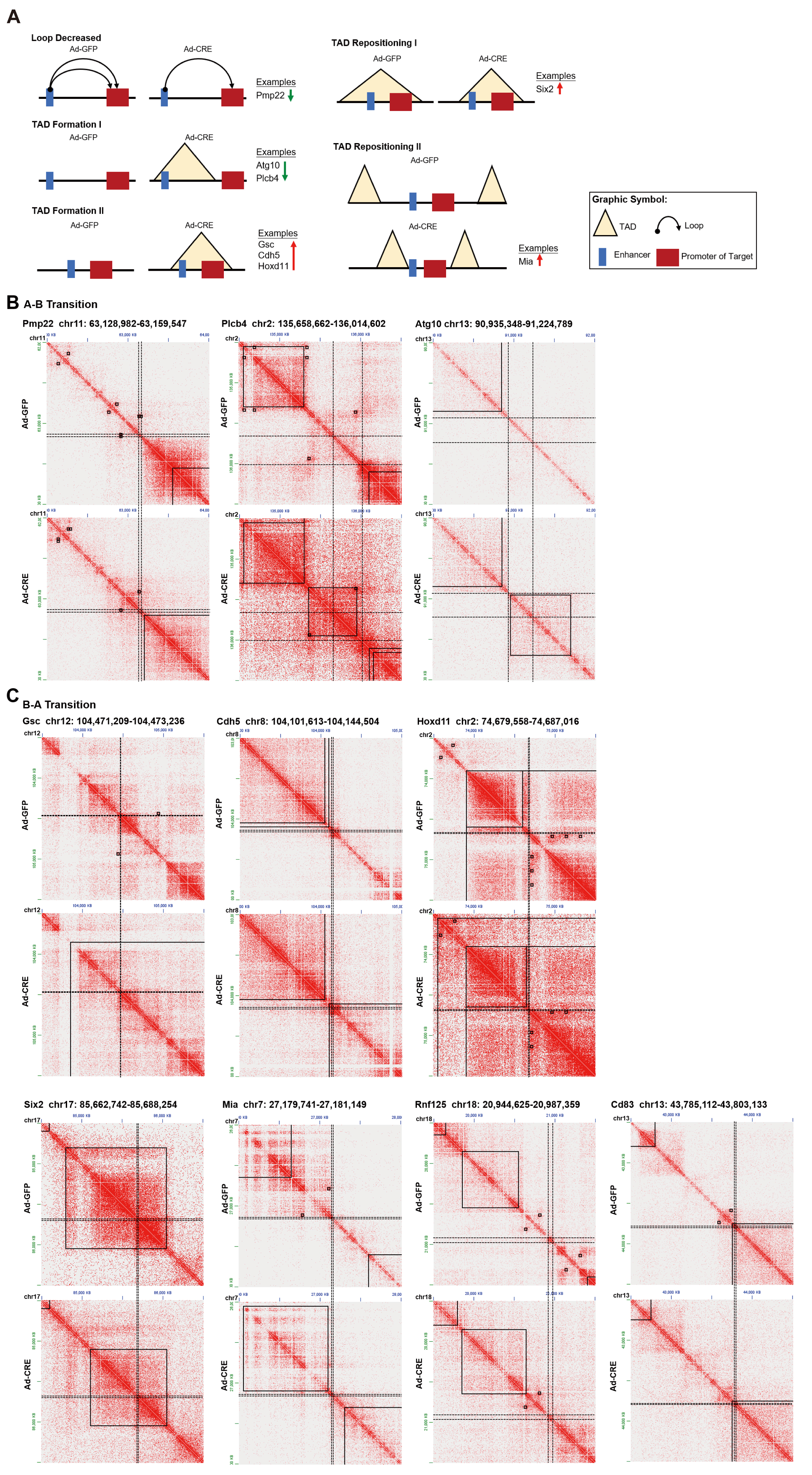

Supplement: Supplementary file 4 — Figure S3 [file 41419_2021_4291_MOESM4_ESM.jpg]

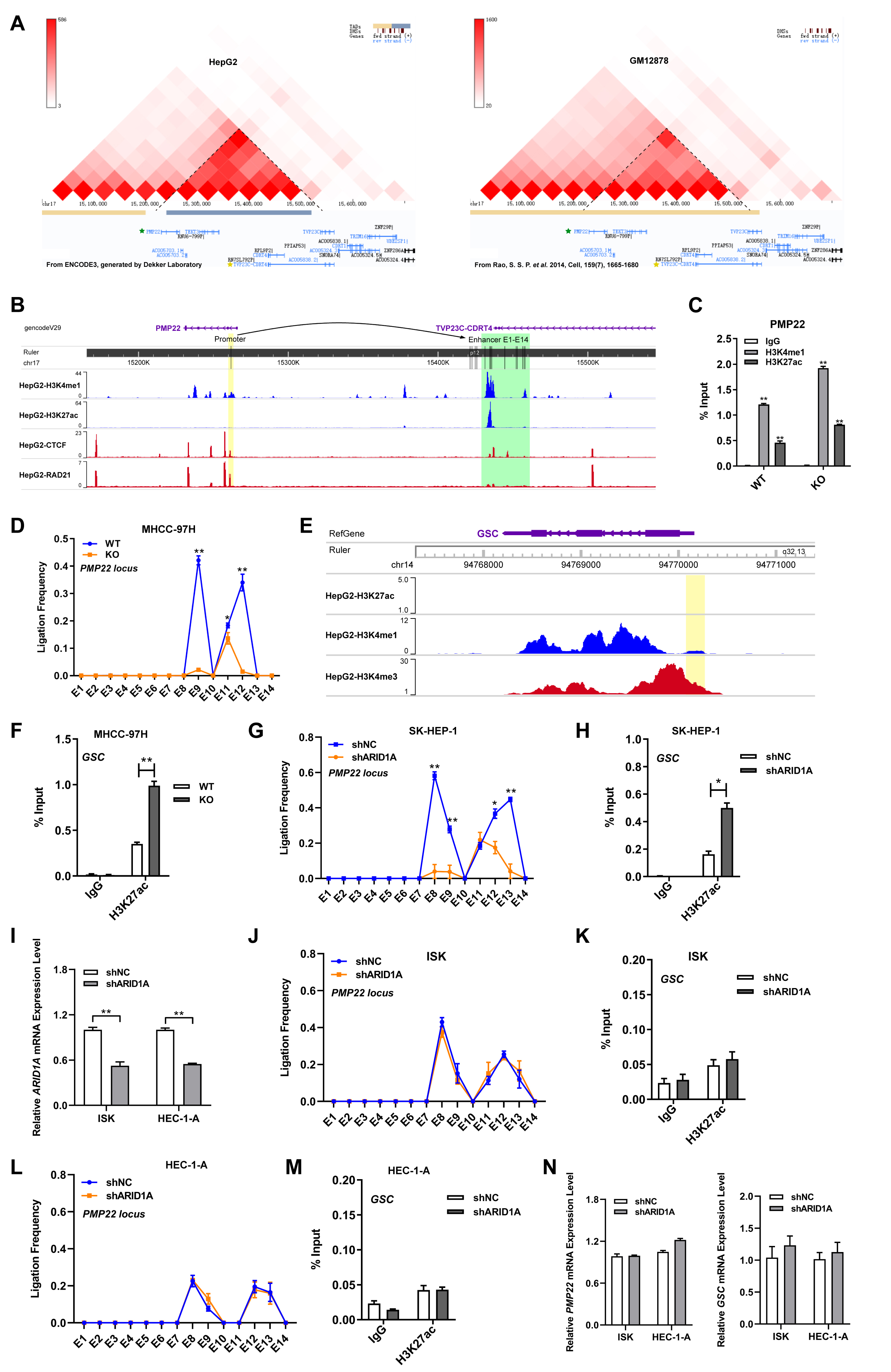

Supplement: Supplementary file 5 — Figure S4 [file 41419_2021_4291_MOESM5_ESM.jpg]

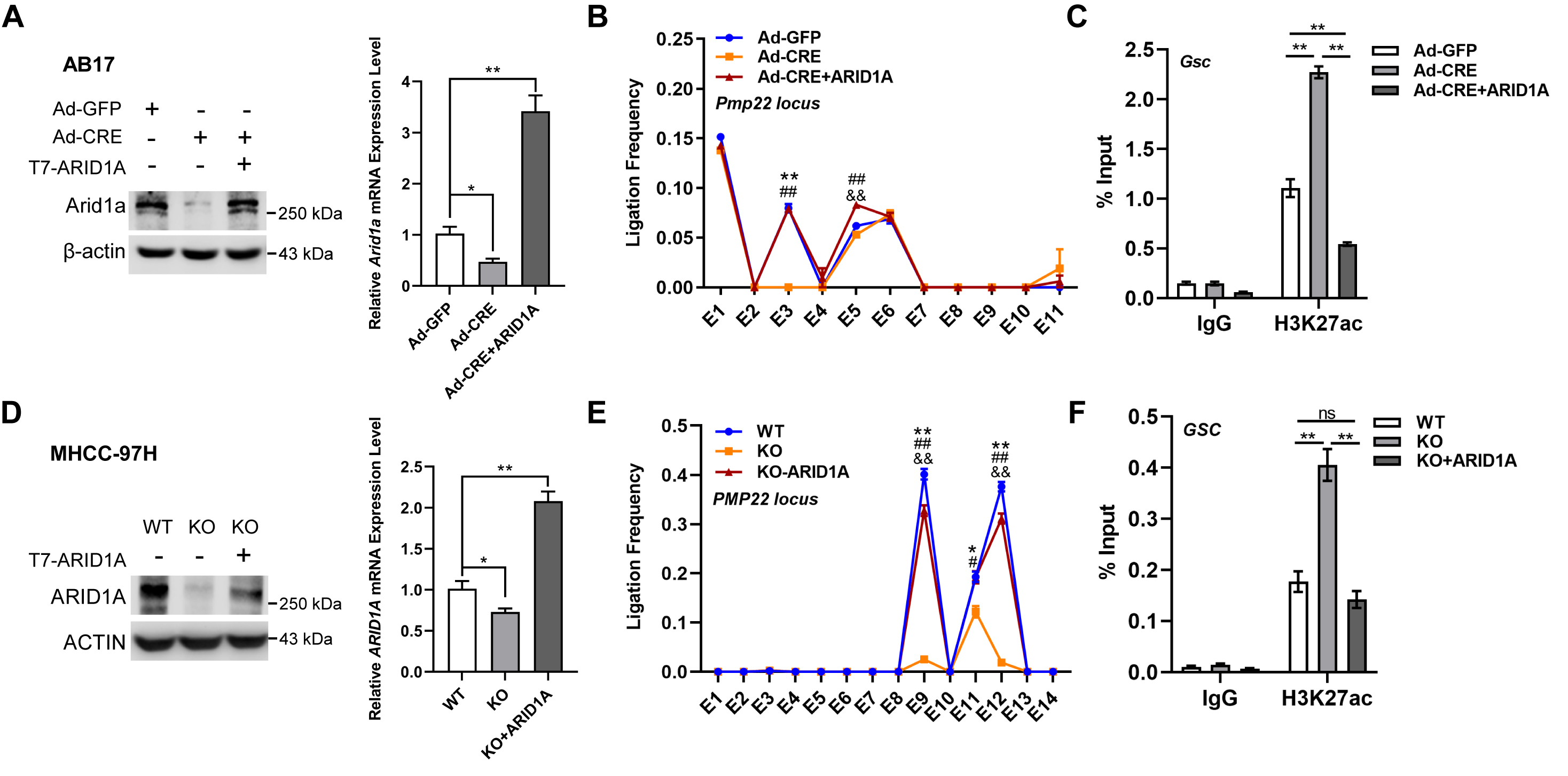

Supplement: Supplementary file 6 — Figure S5 [file 41419_2021_4291_MOESM6_ESM.jpg]

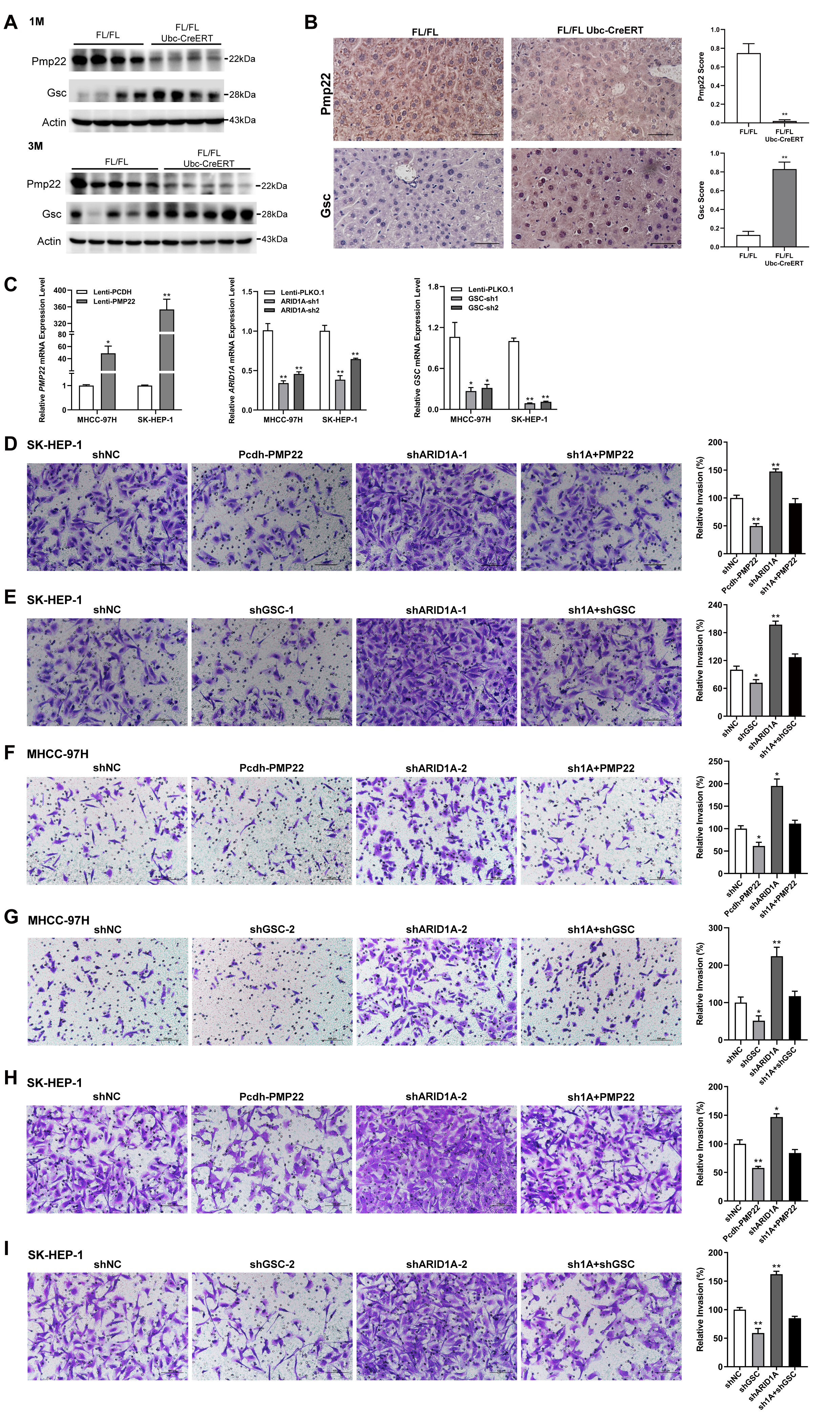

Supplement: Supplementary file 7 — Figure S6 [file 41419_2021_4291_MOESM7_ESM.jpg]
