# Supplementary material for: ARID1A deficiency weakens BRG1-RAD21 interaction that jeopardizes chromatin compactness and drives liver cancer cell metastasis
Source: Cell Death Dis. 2021 Oct 23;12(11):990. doi: 10.1038/s41419-021-04291-6 (PMC8542038; doi:10.1038/s41419-021-04291-6)

Figure 1A

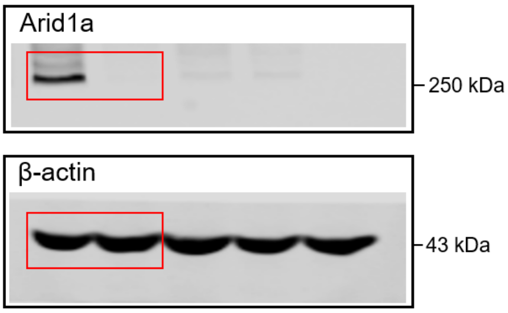

Figure 2A

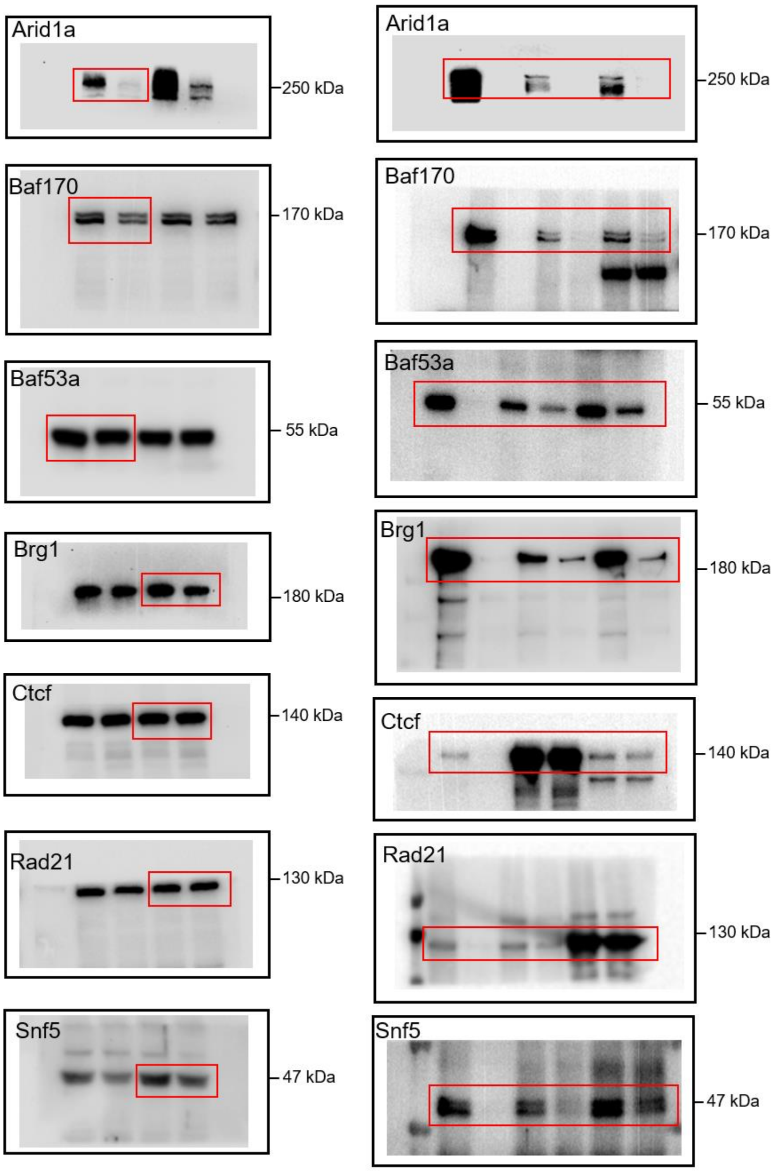

Figure 2B

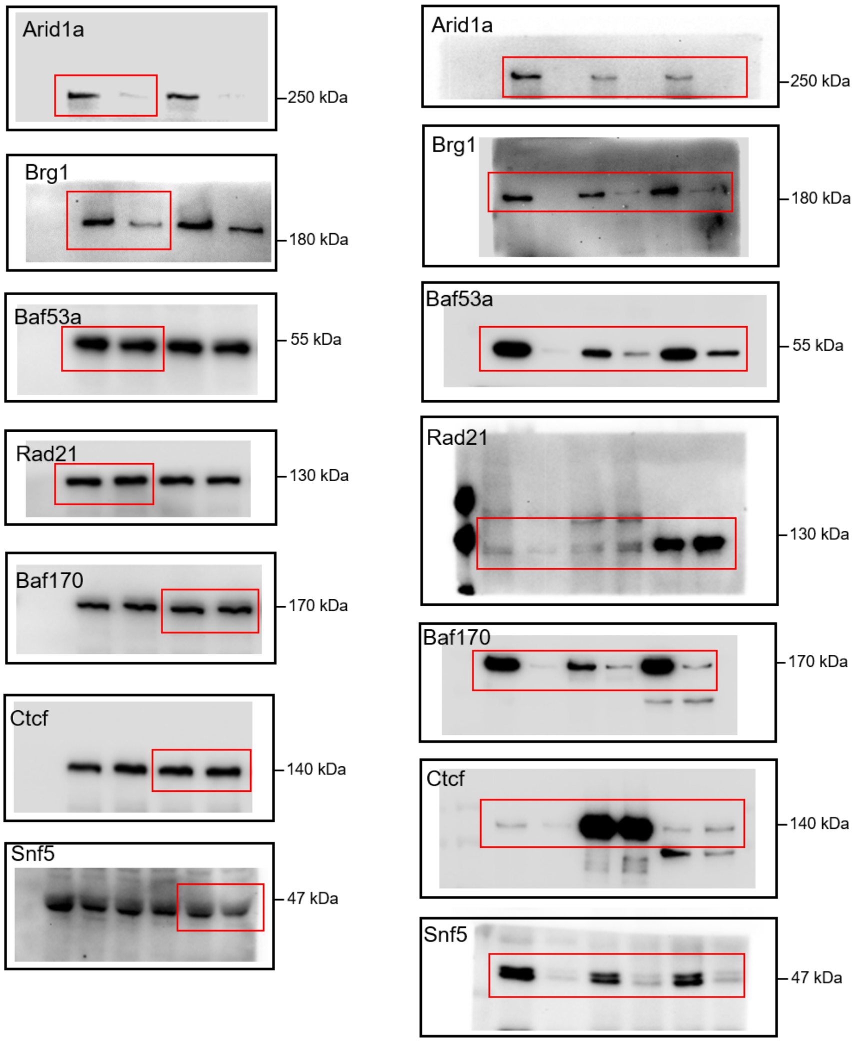

Arid1a
250 kDa
Brg1
180 kDa
Baf53a
55 kDa
Rad21
130 kDa
Baf170
170 kDa
Ctcf
140 kDa
Snf5
47 kDa
Arid1a
250 kDa
Brg1
180 kDa
Baf53a
55 kDa
Rad21
130 kDa
Baf170
170 kDa
Ctcf
140 kDa
Snf5
47 kDa

Figure 2C

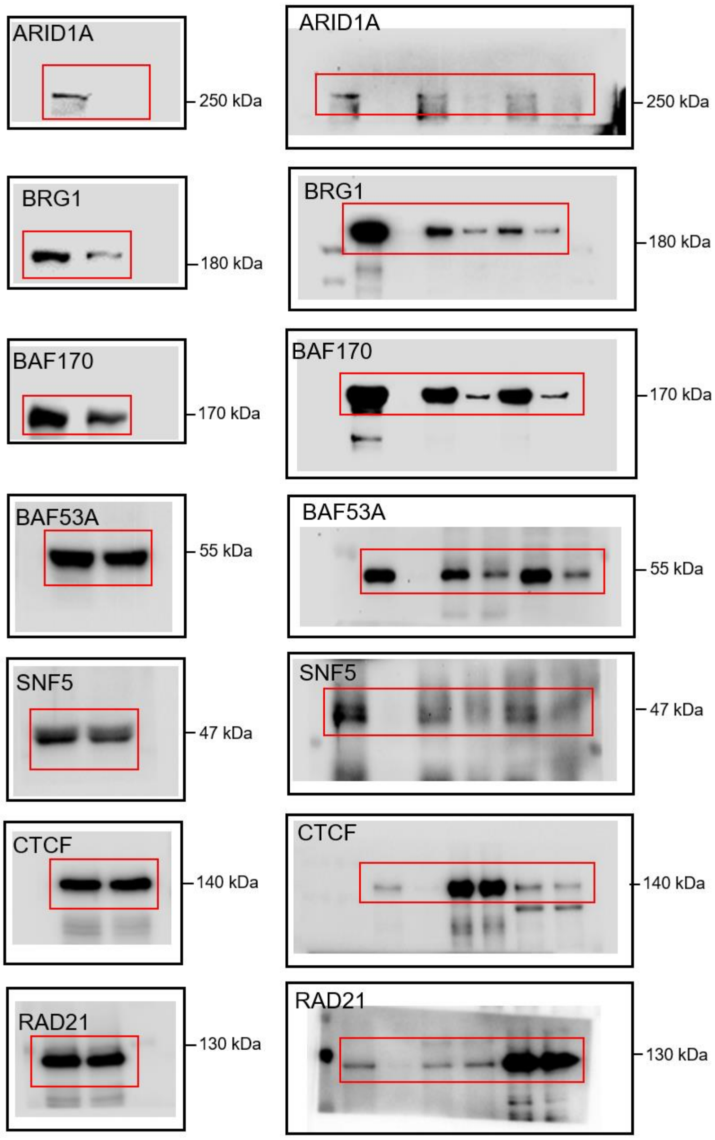
ARID1A
250 kDa
ARID1A
250 kDa
BRG1
180 kDa
BRG1
180 kDa
BAF170
170 kDa
BAF170
170 kDa
BAF53A
55 kDa
BAF53A
55 kDa
SNF5
47 kDa
SNF5
47 kDa
CTCF
140 kDa
CTCF
140 kDa
RAD21
130 kDa
RAD21
130 kDa

Figure 2D

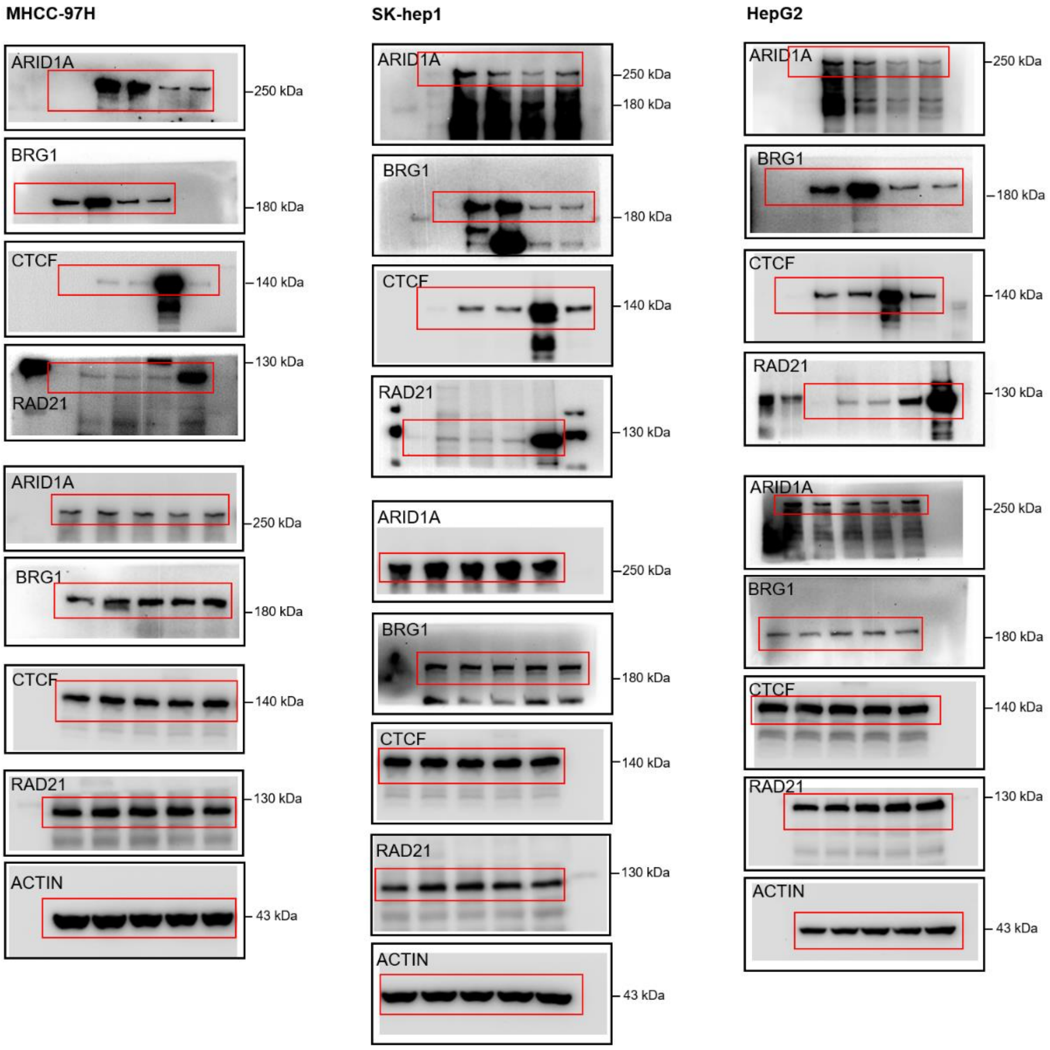
MHCC-97H
SK-hep1
HepG2
ARID1A
BRG1
CTCF
RAD21
ACTIN
250 kDa
180 kDa
140 kDa
130 kDa
43 kDa

Figure 2E

ARID1A-CTCF
IP: HA

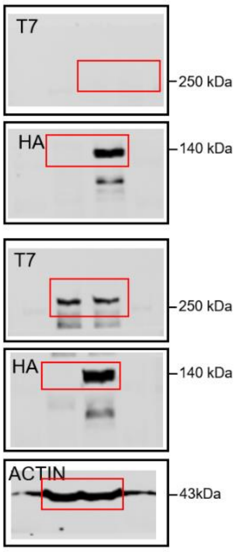

ARID1A-CTCF
IP: T7

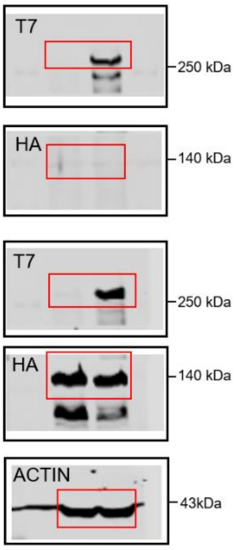

ARID1A-RAD21
IP: HA

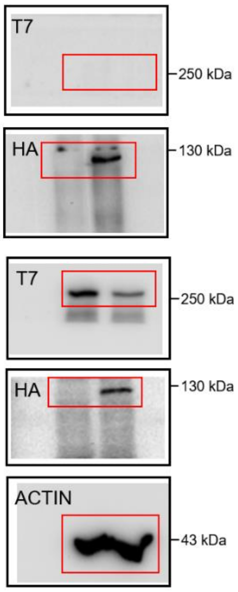

ARID1A-RAD21
IP: T7

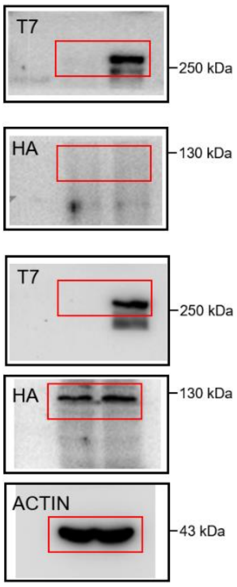

Figure 2F

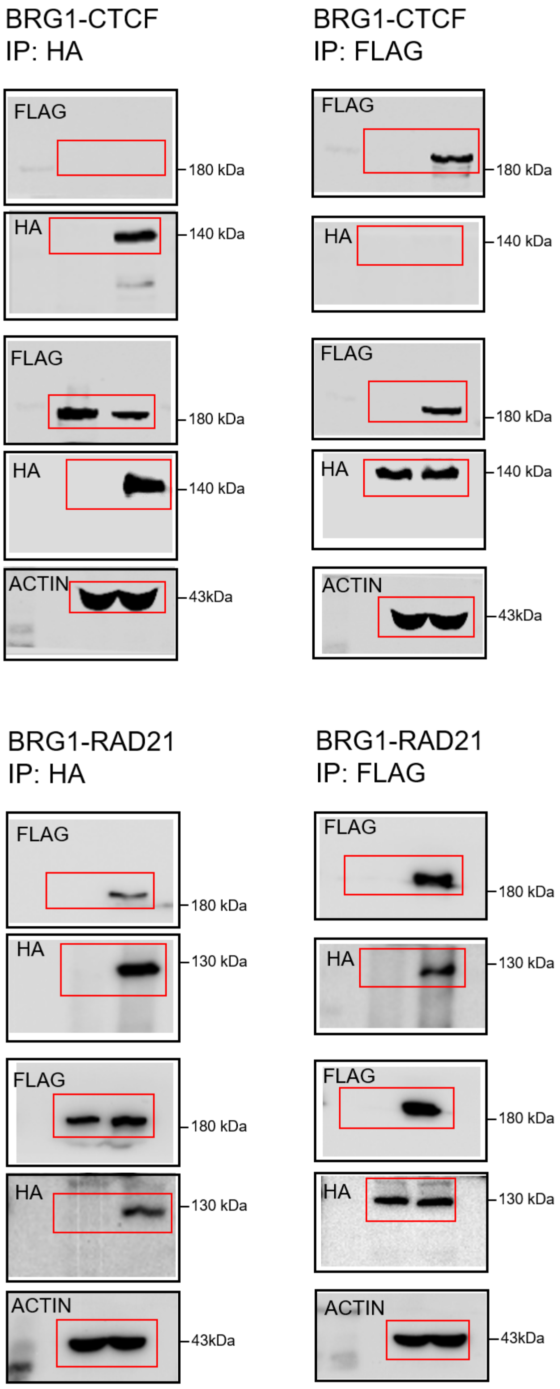
BRG1-CTCF
IP: HA
FLAG
180 kDa
HA
140 kDa
FLAG
180 kDa
HA
140 kDa
ACTIN
43kDa
BRG1-CTCF
IP: FLAG
FLAG
180 kDa
HA
140 kDa
FLAG
180 kDa
HA
140 kDa
ACTIN
43kDa
BRG1-RAD21
IP: HA
FLAG
180 kDa
HA
130 kDa
FLAG
180 kDa
HA
130 kDa
ACTIN
43kDa
BRG1-RAD21
IP: FLAG
FLAG
180 kDa
HA
130 kDa
FLAG
180 kDa
HA
130 kDa
ACTIN
43kDa

Figure 2G

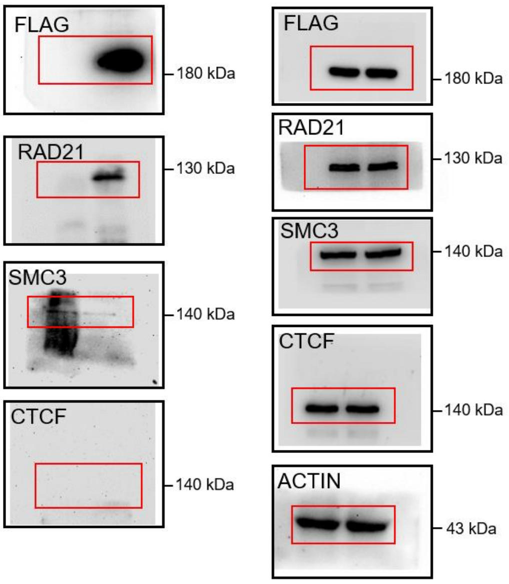

Figure 2H

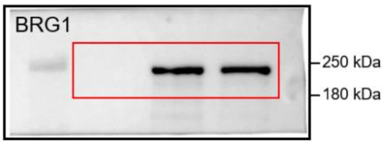

Figure 3A

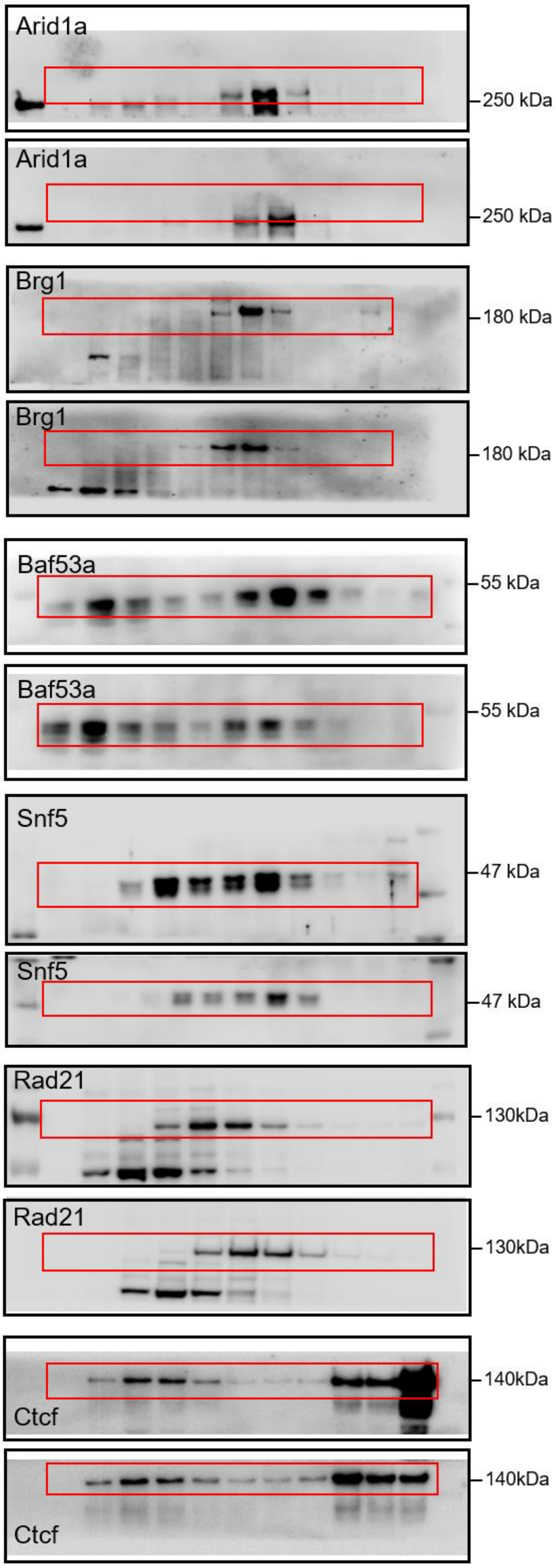
Arid1a
250 kDa
Arid1a
250 kDa
Brg1
180 kDa
Brg1
180 kDa
Baf53a
55 kDa
Baf53a
55 kDa
Snf5
47 kDa
Snf5
47 kDa
Rad21
130kDa
Rad21
130kDa
140kDa
Ctcf
140kDa
Ctcf

Figure 3B

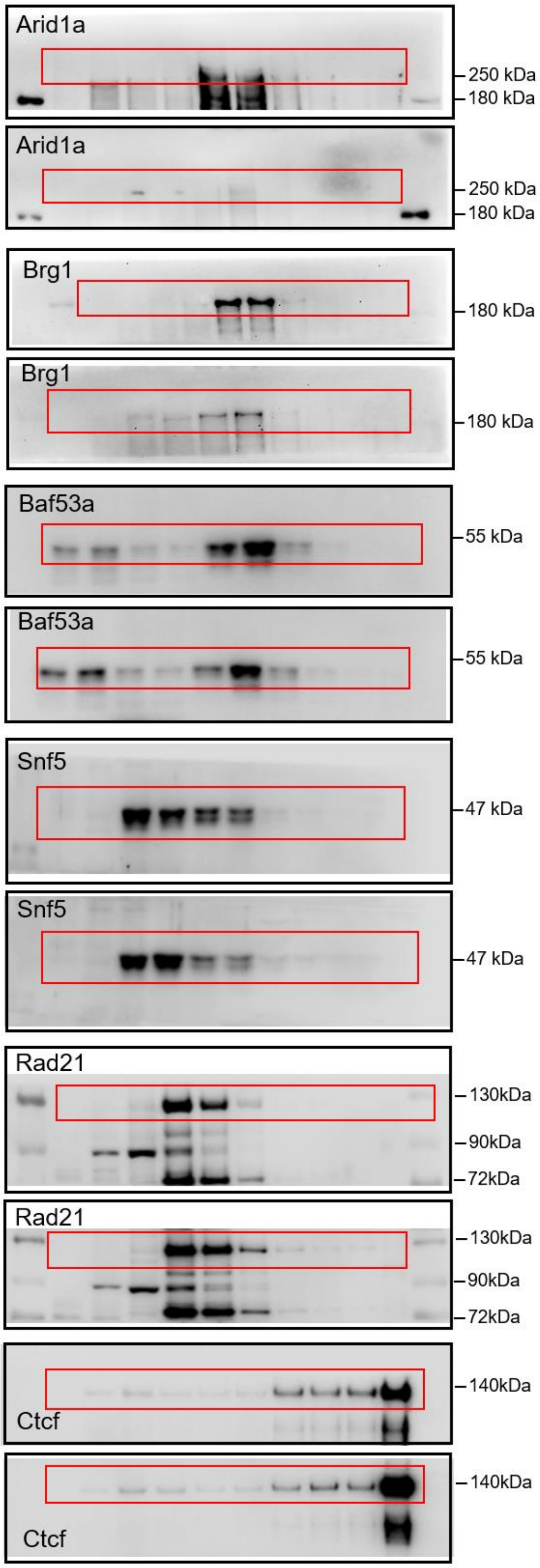

Arid1a
250 kDa
180 kDa
Arid1a
250 kDa
180 kDa
Brg1
180 kDa
Brg1
180 kDa
Baf53a
55 kDa
Baf53a
55 kDa
Snf5
47 kDa
Snf5
47 kDa
Rad21
130kDa
90kDa
72kDa
Rad21
130kDa
90kDa
72kDa
140kDa
Ctcf
140kDa
Ctcf

Figure 3C

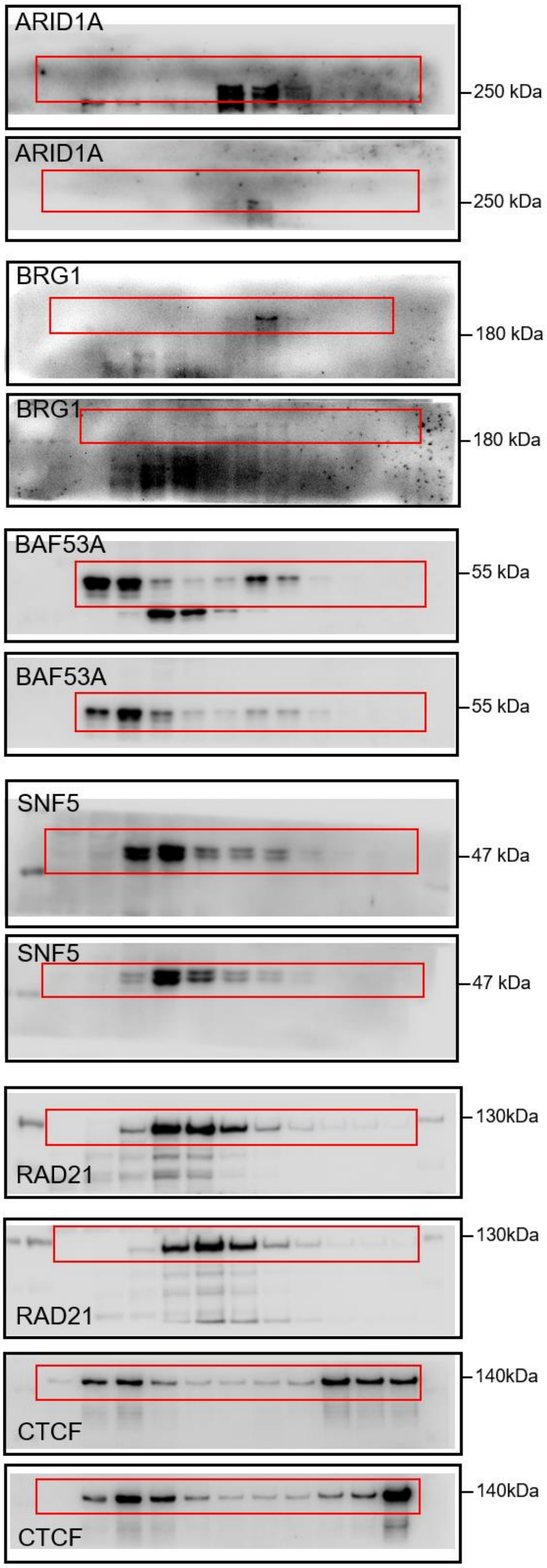
ARID1A
250 kDa
ARID1A
250 kDa
BRG1
180 kDa
BRG1
180 kDa
BAF53A
55 kDa
BAF53A
55 kDa
SNF5
47 kDa
SNF5
47 kDa
130kDa
RAD21
130kDa
RAD21
140kDa
CTCF
140kDa
CTCF

Figure 3D

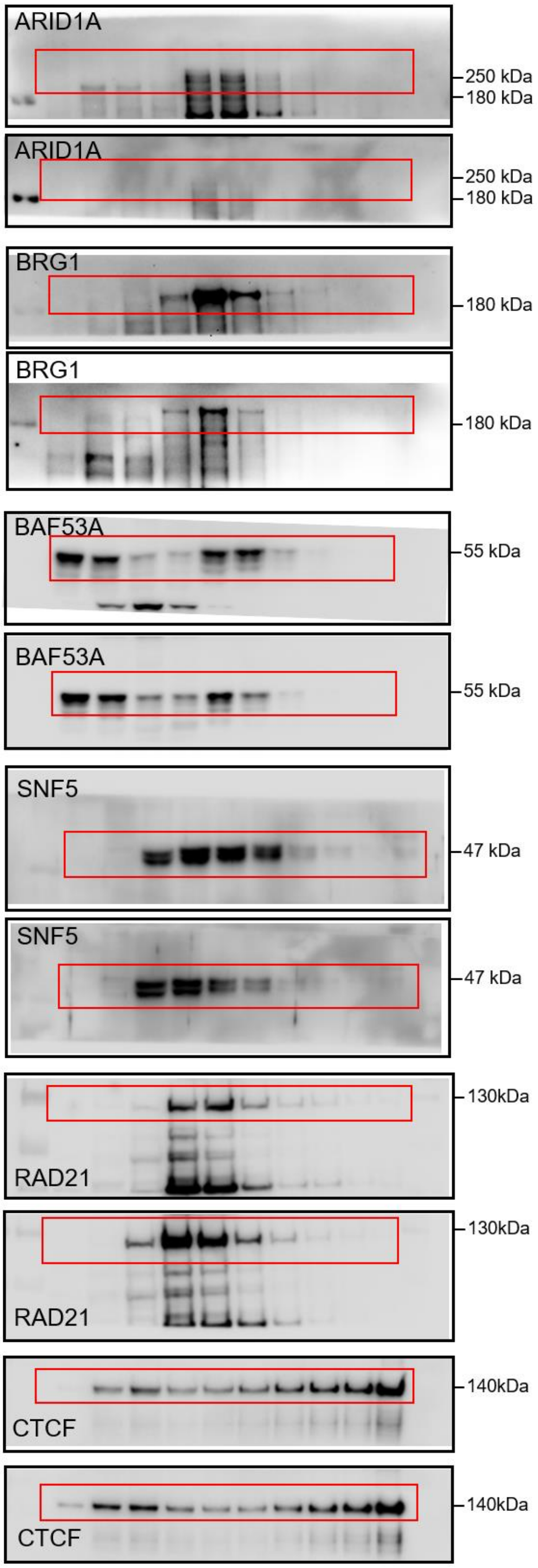
ARID1A
250 kDa
180 kDa
ARID1A
250 kDa
180 kDa
BRG1
180 kDa
BRG1
180 kDa
BAF53A
55 kDa
BAF53A
55 kDa
SNF5
47 kDa
SNF5
47 kDa
130kDa
RAD21
130kDa
RAD21
140kDa
CTCF
140kDa
CTCF

Figure 3E

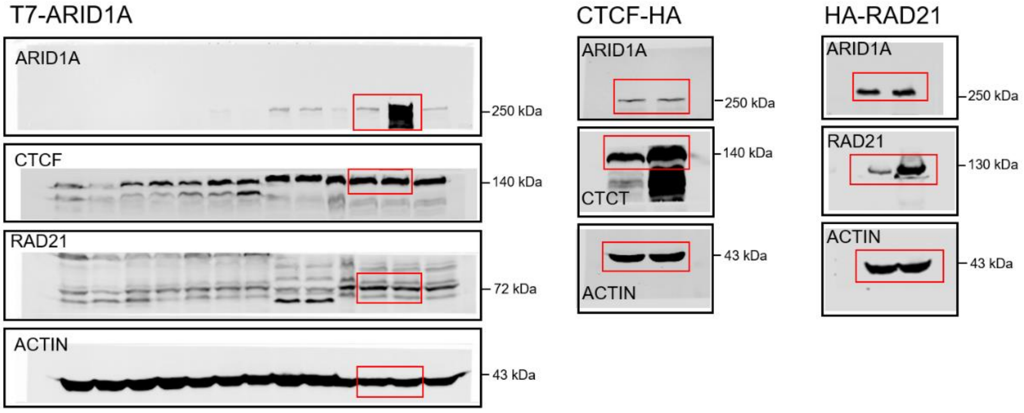
T7-ARID1A
ARID1A
250 kDa
CTCF
140 kDa
RAD21
72 kDa
ACTIN
43 kDa
CTCF-HA
ARID1A
250 kDa
140 kDa
CTCT
43 kDa
ACTIN
HA-RAD21
ARID1A
250 kDa
RAD21
130 kDa
ACTIN
43 kDa

Figure 3F

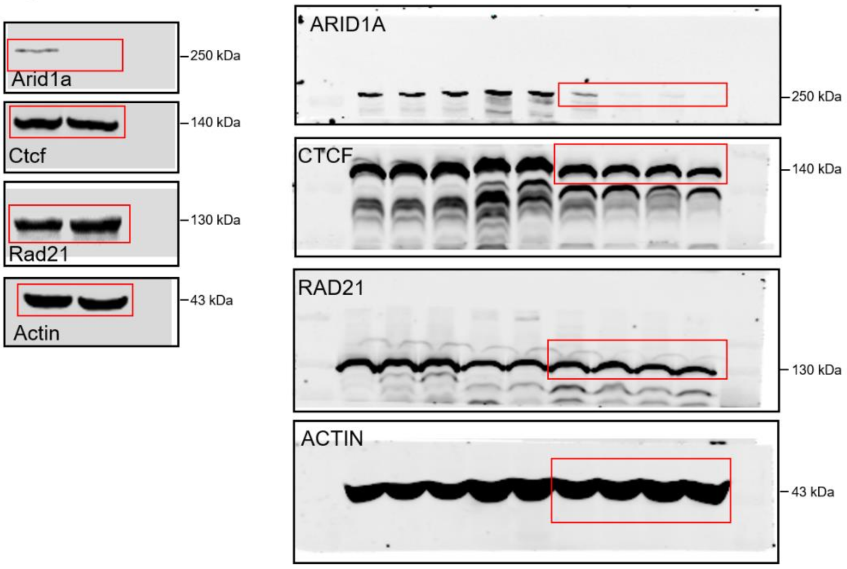
Arid1a
250 kDa
Ctcf
140 kDa
Rad21
130 kDa
Actin
43 kDa
ARID1A
250 kDa
CTCF
140 kDa
RAD21
130 kDa
ACTIN
43 kDa

Figure 6A

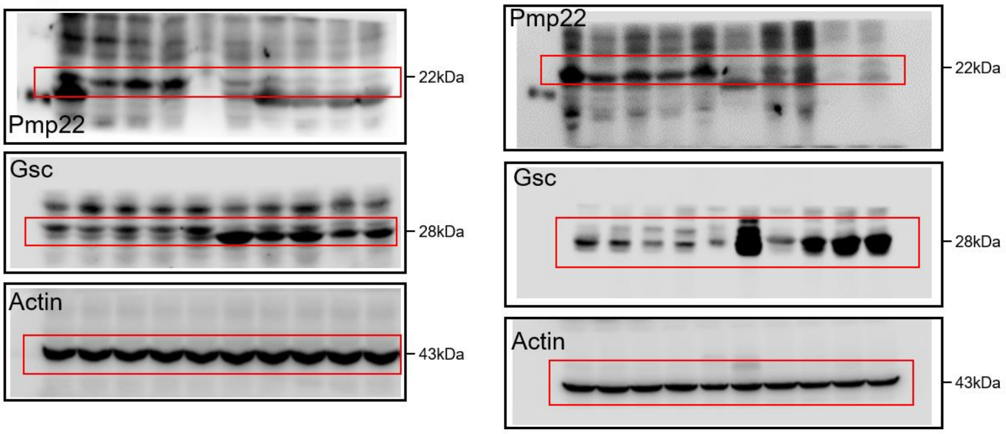
Pmp22
22kDa
Gsc
28kDa
Actin
43kDa
Pmp22
22kDa
Gsc
28kDa
Actin
43kDa

Figure S5A

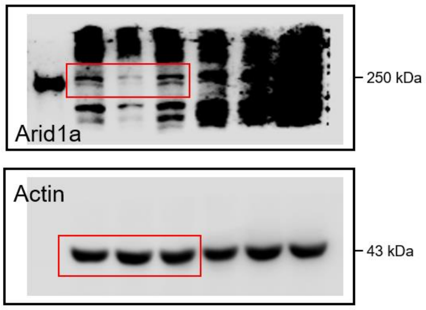

Figure S5D

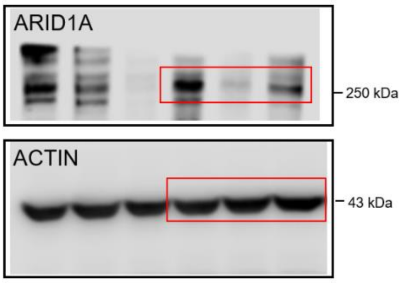

Figure S6A

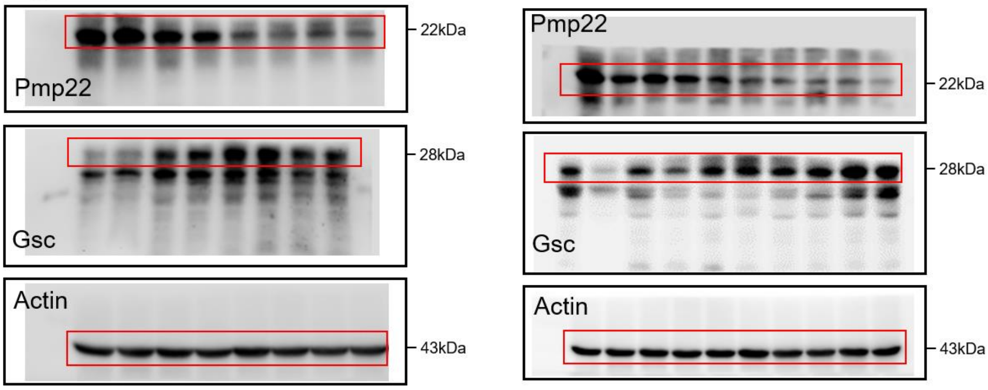

Supplement: Supplementary file 8 — Figure S7 [file 41419_2021_4291_MOESM8_ESM.pdf]
